# Exploiting a targeted resistome sequencing approach in assessing antimicrobial resistance in retail foods

**DOI:** 10.1186/s40793-023-00482-0

**Published:** 2023-03-29

**Authors:** Julie A. Shay, Laura S. E. Haniford, Ashley Cooper, Catherine D. Carrillo, Burton W. Blais, Calvin Ho-Fung Lau

**Affiliations:** Ottawa Laboratory (Carling), Canadian Food Inspection Agency, Ottawa, ON Canada

**Keywords:** Resistome, Microbiome, Metagenomics, Target-capture sequencing, AMR surveillance, Method development, Foodborne microbial hazards

## Abstract

**Background:**

With the escalating risk of antimicrobial resistance (AMR), there are limited analytical options available that can comprehensively assess the burden of AMR carried by clinical/environmental samples. Food can be a potential source of AMR bacteria for humans, but its significance in driving the clinical spread of AMR remains unclear, largely due to the lack of holistic-yet-sensitive tools for surveillance and evaluation. Metagenomics is a culture-independent approach well suited for uncovering genetic determinants of defined microbial traits, such as AMR, present within unknown bacterial communities. Despite its popularity, the conventional approach of non-selectively sequencing a sample’s metagenome (namely, shotgun-metagenomics) has several technical drawbacks that lead to uncertainty about its effectiveness for AMR assessment; for instance, the low discovery rate of resistance-associated genes due to their naturally small genomic footprint within the vast metagenome. Here, we describe the development of a targeted resistome sequencing method and demonstrate its application in the characterization of the AMR gene profile of bacteria associated with several retail foods.

**Result:**

A targeted-metagenomic sequencing workflow using a customized bait-capture system targeting over 4,000 referenced AMR genes and 263 plasmid replicon sequences was validated against both mock and sample-derived bacterial community preparations. Compared to shotgun-metagenomics, the targeted method consistently provided for improved recovery of resistance gene targets with a much-improved target detection efficiency (> 300-fold). Targeted resistome analyses conducted on 36 retail-acquired food samples (fresh sprouts, n = 10; ground meat, n = 26) and their corresponding bacterial enrichment cultures (n = 36) reveals in-depth features regarding the identity and diversity of AMR genes, most of which were otherwise undetected by the whole-metagenome shotgun sequencing method. Furthermore, our findings suggest that foodborne *Gammaproteobacteria* could be the major reservoir of food-associated AMR genetic determinants, and that the resistome structure of the selected high-risk food commodities are, to a large extent, dictated by microbiome composition.

**Conclusions:**

For metagenomic sequencing-based surveillance of AMR, the target-capture method presented herein represents a more sensitive and efficient approach to evaluate the resistome profile of complex food or environmental samples. This study also further implicates retail foods as carriers of diverse resistance-conferring genes indicating a potential impact on the dissemination of AMR.

**Supplementary Information:**

The online version contains supplementary material available at 10.1186/s40793-023-00482-0.

## Background

The increased global awareness of antimicrobial resistance (AMR) has led to the recognition that AMR is an imminent risk to human and veterinary medicine requiring critical attention. In addition to posing a clinical challenge in healthcare, AMR has emerged as a socio-economical issue impacting countries of all income levels. According to the earlier O’Neill report, AMR is projected to claim 10 million human lives and cost the worldwide economy $100 trillion dollars by 2050 if no significant action is taken to tackle this crisis [1]. Recent findings from the first comprehensive assessment of the global AMR burden (2019) further reveal a grand total of 4.95 million deaths associated with bacterial AMR, of which 1.27 million are directly attributable to resistance [2]. In retrospect, drug-resistant infections have caused more deaths worldwide than HIV/AIDS and malaria combined, with only COVID-19 and tuberculosis causing more casualties from infectious diseases [3]. To better address and manage this serious threat to human health, systematic and multi-sectorial surveillance data on the prevalence of AMR and its transmission are imperative, as the well-being of humans, animals, and the environment are intertwined under the “One Health” concept [4]. One of the main conduits of human exposure to AMR organisms and genetic determinants of resistance harbored among the animal and environmental domains is the food production system. While recent efforts have focused on examining the dynamics and distribution of AMR in cropping [5–9] and livestock [10–15] systems under various agricultural practices, relatively little is known about the overall AMR burden associated with finished food products being sold at retail outlets, with which consumers come directly into contact through cross-contamination or consumption of uncooked products.

With rapid advancements and improved affordability of massively-parallel sequencing technology (for example Illumina’s sequencing-by-synthesis platform), it is no longer an arduous task to perform high-resolution genetic analyses on (meta)genomic DNA samples derived from bacterial cultures or environmental samples—even for small molecular biology laboratories with limited resources. Mirroring the whole-genome shotgun sequencing technique, the so-called “shotgun-metagenomics” generally refers to a holistic, culture-independent approach of sequencing the collective genome (i.e. metagenome) of a bacterial community carried by the sample of interest. The key strength of shotgun metagenomics is its ability to generate a fully scalable amount of genomic information specific to the sample’s microbiota that can be readily mined using bioinformatics in order to address complex (micro-)biological and ecological questions. Yet, despite the declining cost of sequencing, whole-metagenome sequencing analysis remains an expensive, time-consuming, and computationally-intensive endeavor. In fact, relative to the average bacterial genome size of ~ 3.6 Mb [16], the microbiomes of human gut [17] and experimental soil [18] are approximately 1,000- and over 20,000- fold larger, respectively. With the current state of technology, it is virtually impossible (or, at least, resource prohibitive) to sequence every nucleotide/gene present in a metagenome while achieving the sequencing depth necessary to attain limits of detection required for characterization of species which represent important but minor components of the microbiome. Additionally, microbial DNA isolation from environmental and clinical samples is most certainly not free from the undesirable co-extraction of genomic material derived from the sample matrix. This non-bacterial DNA can take up a substantial amount of sequencing resources without returning any data of analytical value. Furthermore, various “wet lab” factors can also come into play when determining the detection sensitivity and specificity of a metagenomic method (for example, the choice of DNA extraction protocol, the amount of sample DNA input, and the sequencing chemistry/platform selection, just to name a few [19]). Taken together, these limitations have brought into question the overall effectiveness of shotgun-metagenomics against scenarios in which identification of low-abundance gene targets is required—for instance, profiling the assemblage of AMR determinants (i.e. the resistome) in metagenomic samples.

As an alternative approach, a targeted-sequencing strategy involving in-solution hybridization of the genomic library to a selection of probes prior to sequencing has been developed [20]. Within the context of metagenomics, this targeted approach makes use of short biotinylated RNA probes complementary to the desired sequence targets as “baits” to facilitate hybridization-directed selective recovery of “targets” in the metagenomic samples [21, 22]. Compared to the conventional non-targeted approach of shotgun-metagenomics, this added target capturing procedure should provide for a more focused, target-sensitive sequencing output, together with better sequencing economy (i.e. less time and cost), given the reduced genomic pool size of post-captured DNA libraries.

In this study, we address the development of a sensitive and efficient metagenomic sequencing method for profiling the resistome of microbial samples. We devised a target-capture sequencing workflow that makes use of a customized bait-capture system specific to a large representative collection of antimicrobial resistance genes (ARGs), with validation of the system’s performance using both mock and sample-derived bacterial community preparations, and compare this approach with the non-targeted whole-metagenome shotgun sequencing method. Furthermore, we demonstrate the utility of this method through the characterization of metagenomic sequencing datasets, enabling determination of the identity and diversity of ARGs recovered from several retail foods of high food-safety concerns (e.g. fresh sprouts and ground meat).

## Methods and materials

*Sample acquisition and preparation* A total of 36 food samples were purchased over the period of September 2018 to February 2019 from chain grocery stores and local butcher shops in Ottawa, Ontario Canada. With the exception of a single frozen ground chicken sample (C1), all ground meat samples were purchased as freshly-prepared or in the form of refrigerated, non-frozen packages. Two ground lamb (L2, L3) and 1 organic ground turkey (T4) samples were labelled as “raised without antibiotics”. Once acquired, fresh and frozen samples maintained in their original packaging were kept overnight at 4 °C to simulate typical food handling practices of general consumers. Three 25-g portions of each sample were then aseptically transferred to separate 55 oz. Whirl-pack® sampling stomacher bags (Thermo Fisher Scientific, Nepean, ON, Canada) and mixed with 225 ml of modified tryptone soy broth (Oxoid, Nepean, ON, Canada) by gently massaging the sample bags by hand. After blending for 1 min at a speed setting “1” in a BagMixer® 400VW (Interscience Laboratories, Woburn, MA, United States), 50 ml of food homogenate filtrates were transferred into individual falcon tubes. The remaining bag contents were then incubated aerobically without agitation at 37 °C overnight (for selective enrichment of potentially significant foodborne bacteria, including those belonging to the *Enterobacteriaceae* family), before collecting 10 ml of the resultant enrichment culture filtrates. To retrieve the sample-associated microbiota, a differential centrifugal approach was employed [22]. Briefly, immediately after collection, the homogenate/culture filtrates were centrifuged at 500 × g for 5 min at 4 °C to precipitate any remaining food particles. The supernatant was then further centrifuged at 13,000 × g for 20 min to pellet the bacteria. After the supernatant was removed and an additional round of centrifugation at 13,000 × g for 5 min, any residual liquid was further removed from the tube and the resultant pellet (which consisted of the sample microbiota) kept at − 20 °C until further downstream processing.

*Metagenomic DNA extraction and library construction* Total bacterial DNA of individual food and cultural enrichment samples were extracted from thawed microbiota pellets (i.e. three per sample) using the DNeasy™ PowerSoil Kit (Qiagen, Mississauga, ON Canada) and a Bead Mill-24 homogenizer (Thermo Fisher Scientific) according to the manufacturer’s instructions. Prior to metagenomic library construction, individual DNA samples originating from the same food/enrichment sample were combined in equal weight quantities. The pooled DNA samples (ranging from 1.5 to 2.4 μg) were then purified using the Genomic DNA Clean & Concentrator -10 kit (Zymo Research, Irvine, CA, United States) and an elution volume of 40 μl. This was followed by further concentrating the purified DNA products to final volume of ~ 15 μl using a Vacufuge Plus centrifuge concentrator (Eppendorf, Mississauga, ON Canada). To construct the sequencing libraries described in this study, NxSeq® AmpFREE Low DNA library kit (Lucigen, Middleton, WI, United States) was used with a standard input DNA amount of 1 μg that was sheared to an average size of ~ 400 bp inside a microTUBE-15 (PN 520,145) using a E220 evolution focused-ultrasonicator (Covaris, Woburn, MA, United States). Unique dual indexing of libraries was achieved using the IDT-Illumina TruSeq UD indexes (Illumina, Vancouver, BC, Canada). Unless otherwise specified, all DNA quantitation was routinely conducted using the fluorescence-based Qubit™ dsDNA HS Assay Kit (Thermo Fisher). Sizing of DNA fragments and library products were performed on a 2100 Bioanalyzer using the High Sensitivity DNA reagent kit (Agilent, Mississauga, ON Canada).

*Bacterial mock community DNA sample *A synthetic bacterial community consisting of 25 ATCC Bacteriology Collection strains and 10 foodborne isolates with diverse AMR profiles was constructed (Additional file 3: Table S1). Genomic DNA of individual strains recovered as single colonies on Brain Heart Infusion Agar (BHI agar) plates was extracted from 1 ml of pure BHI broth cultures (overnight incubation at 30 °C with mild agitation) using the DNeasy® Blood Tissue Kit (Qiagen) according to the manufacturer’s instruction. Duplicated mock community DNA samples (hereafter refer to as MC-A and MC-B) were prepared by combining equal amount of genomic DNA isolated from each component of the mock community. Again, 1 μg of the pooled, vacufuge-concentrated mock community DNA was fragmented and converted into a sequencing library as described above.

*Targeted- and whole-metagenome sequencing* For targeted metagenomic sequencing, myBaits® Custom DNA-Seq kits consisting of approximately 60,000 unique biotinylated RNA probes (with an average probe size of 80 nt and 50% sequence overlapping between adjacent probes to give an approximate 2 × coverage of every targeted base) that were complementary to 4,272 targeted gene/genetic marker sequences (Additional file 1) covering a total target space of 4.3 Mbp were designed and manufactured by Arbor Bioscience (Ann Arbor, MI, USA). Hybridization-based capturing and enrichment of targeted sequences present in the dual-indexed libraries (as described above) were performed in accordance to the myBaits® Custom kit’s manual v4.01, followed by Illumina paired-end sequencing. Generally, pre-capture library amplification was performed in individual 50-μl reaction mixtures containing 100 ng of library DNA, primers (reamp-P5/reamp-P7 [23]) at 500 nM final concentration, and 1X KAPA HiFi HotSart ReadyMix (Roche, Laval, QC, Canada). The mixtures were heated for 2 min at 98 °C, followed by 9 cycles of 20 s at 98 °C, 30 s at 60 °C, and 45 s at 72 °C, before finishing with 5 min at 72 °C. The amplified libraries were subsequently purified using 90 μl of Agencourt AMPure XP beads (Beckman, Mississauga, ON, Canada) and eluted with nuclease-free ultrapure water. For individual in-solution hybridization reactions, equal amount (800 ng each) of amplified DNA derived from two sample libraries was pooled and vacufuge-concentrated to 7 μl, before combining with other reaction components. After 20 h of hybridization at 65 °C, the probe-target complexes were allowed to bind to streptavidin-coated magnetic beads for 15 min, and the unbound non-target DNA removed subsequently via three rounds of washing with the provided wash buffer. The bead-bound, target-captured libraries were resuspended in 10 mM Tris–Cl and 0.05% TWEEN-20 (pH 8.0) with a final volume of 30 μl. Post-capture amplification was performed using the exact conditions as described above, except 15 μl of the bead-bound library DNA was included as template and that a total of 12 amplification cycles were used. After magnetic removal of the streptavidin-coated beads, the supernatant containing the enriched libraries was purified using Agencourt AMPure XP beads and eluted with 10 mM Tris–Cl (pH 8.0). For negative controls, library-free amplification and hybridization reactions were routinely included and prepared using nuclease-free ultrapure water in lieu of library DNA sample. Multiplex high-throughput sequencing of the target-enriched libraries was performed on either the Illumina NextSeq 500 platform using the mid-output v2.5 kits (300-cycle) and a loading concentration of 1.5 pM with 1% PhiX spike-in, or, the Illumina HiSeq 4000 platform (Genome Quebec Expertise and Services Center, Montreal, QC, Canada), to generate 2 × 150 bp paired-end sequences with a targeted output of 10–20 million raw reads per sample.

For whole-metagenome shotgun sequencing, metagenomic libraries prepared as described above but without target-capture were quality-verified and sequenced on the Illumina platforms NovaSeq 6000 (S4 flow cell) or HiSeq 4000 (Genome Quebec Expertise and Services Center), to generate 2 × 150 bp paired-end sequences with a targeted output of 50–100 million raw reads per sample.

*16S ribosomal RNA gene amplicon libraries construction and sequencing* An amplicon sequencing workflow utilizing the hypervariable V3-V4 regions of bacterial 16S rRNA gene as taxonomic classifier [24] was employed to estimate the composition of microbial community recovered from food and enrichment culture samples. Briefly, 12.5 ng of sample DNA was used as template for individual 25-μl PCR reactions containing each of the V3 and V4 primers with overhang adaptor sequence at 200 nM and 1X KAPA HiFi Hotstart Ready Mix (Roche) according to the Illumina protocol. Control reactions devoid of template were included and the reaction products processed (and ultimately sequenced) as individual control libraries alongside other sample libraries. After purification of the 16S rRNA amplicons with Agencourt AMPure XP beads (Beckman), dual-indexing of individual reaction products was achieved by using Nextera® XT index Kit v2 Set A (Illumina). Indexed amplicon libraries were purified by use of Agencourt AMPure XP beads, normalized and pooled at a final concentration of 4 nM. High-throughput sequencing of the amplicon libraries was performed on a MiSeq system (Illumina) using the MiSeq reagent kit v3 (600-cycle) and a loading concentration of 4 pM with 5% PhiX spike-in, to generate 2 × 300 bp paired-end sequences with a targeted output of approximately 200,000 raw reads per sample.

*Bioinformatics analysis* Bait-capture target sequence clusters were originally generated using CD-HIT [25] using a 90% identity cut-off. Antimicrobial resistance (AMR) target genes, plasmid replicon sequences, and target genes identified as fusion genes were each clustered separately. For AMR gene annotations, antibiotic classes were initially extracted from AMRFinderPlus [26], and mechanisms for many genes were extracted from the CARD database ver. 3.0.3 [27]. For replicon sequence annotation, groupings of these plasmid-derived sequences based on their incompatibility groups, Pfam domain, and sequence similarity were retrieved from the PlasmidFinder database (accessed in October 2017) [28]. Annotations for both the AMR and plasmid replicon targets underwent multiple rounds of manual curation to check for consistency and errors.

For the detection of sequence targets from both the targeted- and the whole-metagenome shotgun sequencing datasets, a reference-based bioinformatics pipeline modeling on AMRPlusPlus ver. 2.0.0 [29] with custom scripts was developed (https://github.com/jashay90/targetdetection). Trimmomatic [30] version 0.38 was used with recommended settings to remove adapters, remove leading and trailing bases below a Phred score of 3, a sliding window to cut reads when the average base quality drops below a Phred score of 15, and dropping reads less than 36 bases long after trimming. Host contamination filtering was performed by aligning reads to host genomes (Additional file 3: Table S2) using BWA-MEM ver. 0.7.17 [31] and removing reads which were mapped in proper pairs. The retained reads were then aligned to the total bait-capture target sequence set using BWA-MEM, with custom python scripts being used subsequently to summarize read counts and gene coverage, and to summarize the results according to predefined gene clusters. Unless otherwise specified, target detection was defined at ≥ 90% coverage across the length of the sequence.

Synthetic whole-metagenome shotgun sequencing datasets of bacterial mock community samples were generated in silico using ART [32]. Equal numbers of in silico reads were generated for each of the 35 component organisms, which were then concatenated and shuffled using the Perl script *fastq-shuffle* ver. 0.9.1 [33]. Similarly, in silico subsampling of reads was performed by shuffling reads from a given sample using fastq-shuffle, and extracting the desired number of sequences from the beginning of each resulting file.

For microbial profiling of bacterial mock community samples based on the whole-metagenome shotgun sequencing dataset, reads passing quality filters from Trimmomatic were classified using MetaPhlAn 2.7.7 with the default MetaPhlAn database [34].

For 16S rRNA microbial profiling sequencing data analysis, QIIME2 [35], an end-to-end microbiome bioinformatics platform, was employed with the DADA2 option for sequence quality control and denoising (Additional file 2), and the SILVA SSU ribosomal RNA sequence database release 132 [36] for taxonomy assignment. To remove primer sequences and any low-quality bases at the 5’ end, the first 27 bases from the forward read and the first 21 bases from the reverse read were discarded. For quality trimming, the forward and reverse reads were truncated at position 277 and 219, respectively, based on manual quality inspection.

*Statistical and data analysis* Unless otherwise specified, the open-source program R ver. 4.0.4 [37] was used for statistical and data computing purposes and all statistics were conducted using normalized read count data. For graphical illustration, R package *ggplot2* ver. 3.3.3 [38] was generally used to construct various heatmaps, stacked bar-charts, box-plots, and ordination plots, with the exception of the Venn diagrams that were plotted by using R package *VennDiagram* ver. 1.6.20 [39]. After removal of any potential contaminants ASV using the “prevalence method” of *decontam* ver. 1.18.0 [40], taxonomic assignment obtained through QIIME2 was further processed by removing any singleton taxa (i.e. single occurrence among all samples), and those assigned to the class “*chloroplast*” and the family “*mitochondria*”, using R packages *qiime2R* ver. 0.99.4 [41] and *phyloseq* ver. 1.34.0 [42], before being visualized by *ggplot2*. Alpha diversity was estimated with the overall richness (observed number of non-redundant AMR genes or replicon types), the Shannon and Simpson diversity Indexes, and the Pielou’s evenness through the R packages *vegan* ver. 2.5–7 [43] and *phyloseq.* To test for statistically significant differences in the alpha diversity data, the non-parametric Kruskal–Wallis one-way ANOVA test followed by Dunn’s post-hoc test, with Bonferroni correction to adjust p values for multiple pairwise comparisons, were performed using the functions “kruskal_test” and “dunn-test” from R package *rstatix* ver. 0.7.0 [44]. Beta diversity was determined through principle coordinates analysis (PCoA) of Bray–Curtis dissimilarity performed through the “vegdist” and “pcoa” functions of R packages *vegan* and *ape* ver. 5.4–1 [45], respectively. Significance of (dis)similarity between and within commodity types were examined by permutational multivariate analysis of variance (PERMANOVA) and analysis of similarities (ANOSIM) using *vegan* functions “adonis” and “anosim” with 999 permutations, and by using R package *pairwiseAdonis* ver. 0.4 [46] function “pairwise.adonis” with Bonferroni correction and 999 permutations for pairwise comparisons between selected types. Procrustes rotation of any two comparing ordination results were done using the “procrustes” and “protest” functions of *vegan*. R package *corrplot* ver 0.84 [47] was used to visualize the correlation matrix and the significant level computed through R functions “cor” and “cor.mtest”, considering only targets and taxa that were present in at least 50% (but not 100%) of the samples to avoid bias due to zero-inflation.

*Data availability* All raw sequencing read files were deposited in NCBI and are available under BioProject accession number PRJNA735263.

## Result

**Fig. 1:**
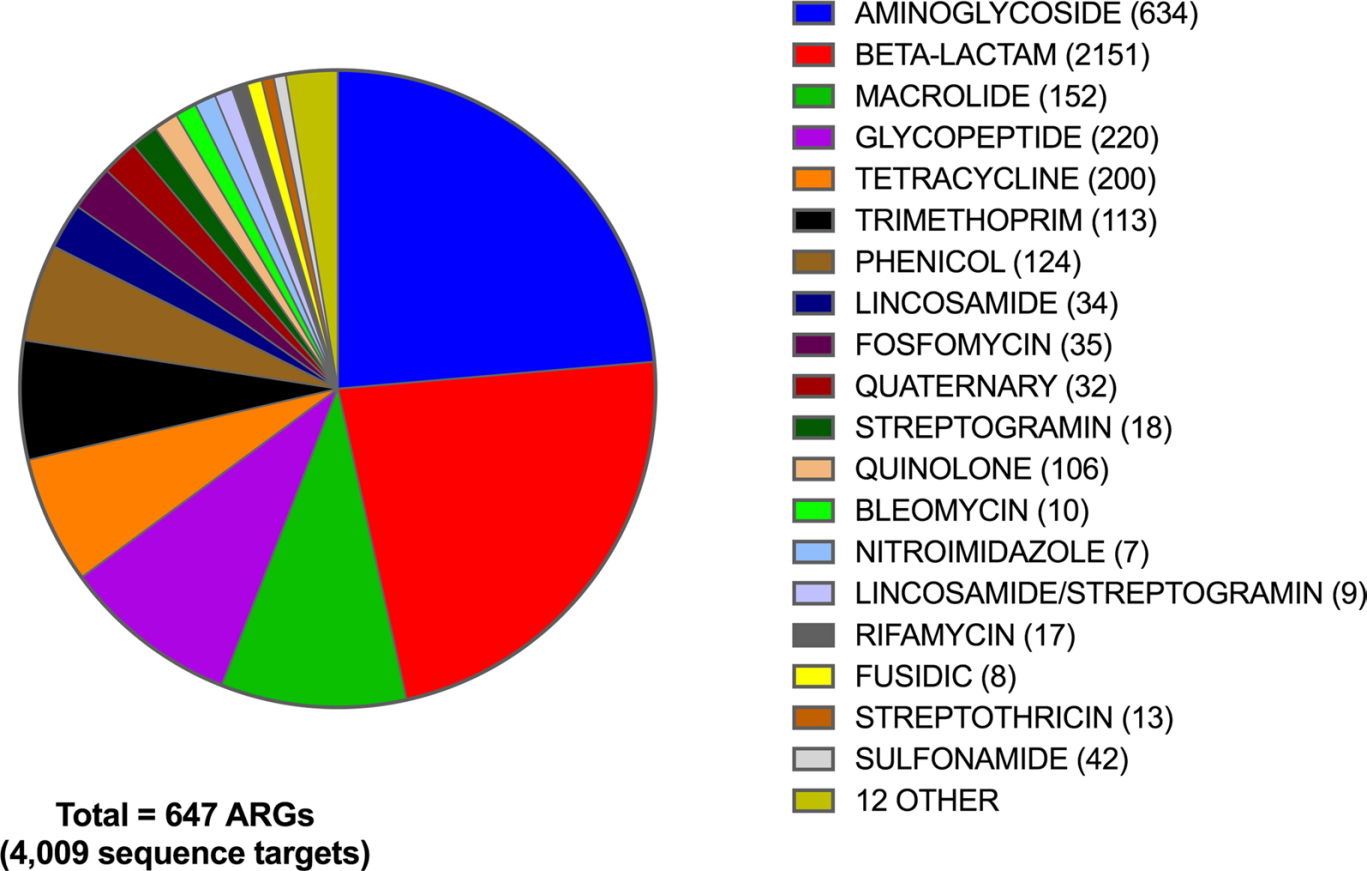
Distribution of antimicrobial resistance gene (ARG) targets included in this study. Number in parentheses indicates the number of non-redundant ARG sequences included for each antibiotic resistance type

**Targets selection and database description **To allow for the comprehensive detection of antimicrobial resistance genes (ARGs) in complex environmental samples, a collection of 4,009 curated DNA sequences obtained from NCBI’s Bacterial Antimicrobial Resistance Reference Gene Database (NCBI bioproject PRJNA313047, original access date: 2017-01-13) was chosen as the core set of targets of a custom-built target capturing system. Identity-based CD-HIT clustering [25] of these target sequences resulted in 1,004 groups of representative sequence (hereafter referred to as “ARG clusters”). Individual ARG clusters encompass genetic sequence variants belonging to one of the 647 non-redundant AMR genes/gene families that can be further classified into 30 different resistance types based on antibiotic classes (Fig. 1). In addition to the ARG targets, a total of 263 replicon sequences, representing 96 different replicon types, were retrieved from the PlasmidFinder database [28] and were included as targets of the same bait-capture system (Additional file 1). Identity-based grouping of these replicon sequences in conjunction with the core ARG targets further generated 180 replicon-specific clusters, hereafter referred to as “plasmid clusters”.

### Performance assessment—microbial mock community

For the purpose of evaluating the target detection efficiency of this customized bait-capture system, a 35-member *in-vitro* mock community sample was subjected to Illumina high-throughput sequencing analyses using targeted (i.e. bait-capture) and non-targeted (i.e. whole-metagenome shotgun) approaches in parallel. While the microbial taxonomic profile derived from the whole-metagenome sequencing data corroborated the mock community sample’s bacterial composition (Additional file 3: Fig. S1), mapping of the 27 million quality-filtered reads generated against the ARG and replicon target sequences only yielded an ‘on-target’ rate of slightly over 0.2% (i.e. ~ 2 out of 1,000 reads were in alignment with the target sequences) (Table 1). With bait-capture, despite the reduced sequencing effort, the on-target rate was drastically improved by over 300-fold to an estimated average of 67% (Table 1).

**Table 1:** Sequencing metrics of mock community sample data generated using whole-metagenome shotgun and bait-capture approaches

Sample annotation	Metagenomics sequencing method^1^	Number of raw read (million)	Number of pass quality-filter read (million)	Number of on-target read (million) ^2^	On-target rate ^3^
MC-A	SH	36.31	26.97	0.05	0.2%
MC-A	T	8.84	6.39	4.19	65.6%
MC-B	T	5.80	4.29	2.93	68.1%

^1^SH, Whole-metagenome shotgun; T, Targeted bait-capture

^2^On-target read refers to sequence read that can be mapped onto at least one of the target sequences

^3^On-target rate is defined as percentage of the pass quality-filter reads that is on-target

**Fig. 2:**
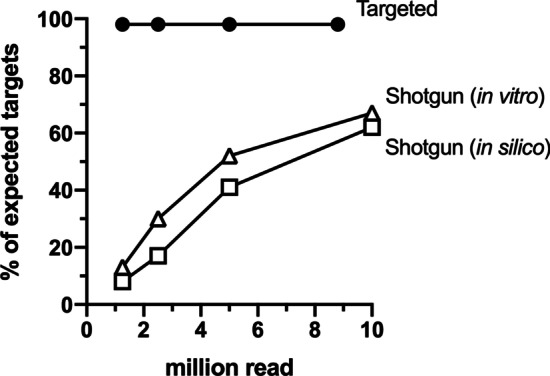
Target recovery by bait-capture (*Targeted*) and whole-metagenome (*Shotgun*) sequencing of defined mock community sample. Percentage values were determined with a ≥ 90% target-coverage detection threshold value applied and based on 105 expected target clusters in total. For clarity, the overlapping targeted-sequencing result from the duplicated mock community sample MC-B was not shown

To compare the sensitivity of target detection by the two metagenomic sequencing methods, their respective sequencing read sets were subsampled to comparable sizes and the corresponding target recovery rate determined over a range of detection threshold ‘cut-off’ values. A coverage-based detection threshold represents the minimal percentage of length of the target gene required to be covered by aligned reads in order to be considered a positive detection, thus, serving as a proxy for detection confidence. Consistent with the in silico simulated shotgun dataset, target detection without bait-capture was dependent on both sequencing depth and detection threshold (Fig. 2, Additional file 3: Table S3). For instance, with a subsampling size of 10 million reads and the most stringent detection cut-off value applied (i.e. ≥ 90% sequence coverage), only 65 of the 105 expected target clusters were recalled (Additional file 3: Table S3). In contrast, all except two of the expected target clusters were recovered from as few as 1.25 million raw reads derived from the target-enriched metagenomic DNA libraries (Fig. 2), and the detection threshold increment had minimal impact on the overall target recovery rate (Additional file 3: Table S3). As such, it is clear that the bait-capture sequencing method not only allows better recovery of the targeted ARGs and plasmid replicons at a higher level of confidence, and it can do so more efficiently compared to the conventional non-targeted approach.

### Resistome analysis of food commodities

To examine the AMR burden associated with a sampling of different food commodities that are generally known to carry high levels of background bacteria, thus increasing the likelihood of AMR bacteria being present, targeted resistome sequencing was conducted with the total DNA extracted from 36 different retail samples of ground meat and fresh sprouts (Additional file 3: Fig. S2), and their corresponding selective-enrichment cultures. Again, the superior ability of the present target capturing system to enrich for sequence targets within metagenomic libraries was fully illustrated by comparing the total read outputs obtained on a subset of these food/enrichment samples with and without bait-capture (Table 2). Among the fifteen randomly-selected samples, the targeted sequencing method attained an average on-target rate of 46.19 ± 4.68% versus 0.13 ± 0.02% by the non-targeted method. While both meat and meat-derived selective-enrichment samples displayed various degrees of non-microbial sequence contamination attributable to host DNA carryover at the sample preparation stage, there was generally a lesser proportion of host-originated reads in the sequencing datasets when bait-capture was applied (i.e. more reads retained for downstream target detection analysis). These, collectively, resulted in more ARG and plasmid clusters being detected from the bait-capture datasets than from the whole-metagenome datasets across all fifteen samples, regardless of food commodity types (Additional file 3: Fig. S3).

**Table 2:** Sequencing metrics of selected food commodity and enrichment culture sample data generated using whole-metagenome shotgun and bait-capture approaches

Sample annotation	Sample type	Metagenomics sequencing method^1^	Number of raw read (million)	Number of pass quality-filter read (million)	Number of read retained after dehosting (million)	Number of on-target read^2^ (million)	Non-host read percentage	On-target rate^3^
B 5	Ground Beef	SH	101.6	97.1	3.2	0.003	3.3%	0.1%
		T	23.0	21.7	2.2	1.4	10.2%	62.8%
	Ground Beef (enrichment)	SH	66.1	62.2	49.8	0.1	80.0%	0.1%
		T	19.6	17.8	17.7	8.7	99.6%	48.9%
B 3	Ground Beef	SH	86.7	82.8	7.1	0.01	8.6%	0.2%
		T	16.6	16.0	2.8	1.6	17.4%	58.5%
	Ground Beef (enrichment)	SH	90.7	86.8	64.2	0.1	73.9%	0.1%
		T	17.9	16.6	16.6	8.3	99.7%	50.0%
C 1	Ground Chicken (enrichment)	SH	84.6	81.3	66.0	0.2	81.1%	0.3%
		T	25.4	23.6	22.5	13.6	95.1%	60.5%
P 3	Ground Pork	SH	100.8	96.2	50.4	0.03	52.4%	0.1%
		T	28.0	25.2	18.0	8.6	71.4%	47.6%
	Ground Pork (enrichment)	SH	81.3	78.1	61.8	0.1	79.1%	0.1%
		T	33.3	29.9	29.9	14.2	99.9%	47.7%
P 2	Ground Pork (enrichment)	SH	83.7	80.3	61.6	0.2	76.7%	0.3%
		T	29.0	27.1	26.3	14.2	97.1%	54.1%
T 1	Ground Turkey (enrichment)	SH	70.0	58.3	58.2	0.1	99.9%	0.1%
		T	31.4	29.4	29.3	16.8	99.7%	57.5%
AS 2	Alfalfa sprouts	SH	89.9	83.8	78.4	0.1	93.5%	0.2%
		T	21.1	19.4	19.3	3.3	99.7%	17.0%
	Alfalfa sprouts (enrichment)	SH	82.1	65.8	65.8	0.1	100.0%	0.2%
		T	29.4	26.9	26.9	15.0	100.0%	56.0%
MBS 1	Mung bean sprouts	SH	64.4	42.7	42.2	0.003	98.7%	0.01%
		T	11.9	11.3	10.9	0.7	96.0%	6.1%
	Mung bean sprouts (enrichment)	SH	69.7	57.8	57.7	0.1	100.0%	0.2%
		T	27.7	25.7	25.6	14.3	99.9%	55.8%
MBS 3	Mung bean sprouts	SH	63.2	52.5	49.6	0.01	94.4%	0.02%
		T	12.6	12.2	10.8	1.6	88.4%	14.4%
	Mung bean sprouts (enrichment)	SH	69.6	56.7	56.7	0.1	100.0%	0.1%
		T	32.4	29.8	29.7	16.6	99.9%	56.0%

^1^SH, Whole-metagenome shotgun; T, Targeted bait-capture

^2^On-target read refers to sequence read that can be mapped onto at least one of the target sequences

^3^On-target rate is defined as percentage of the non-host reads that is on-target

**Fig. 3:**
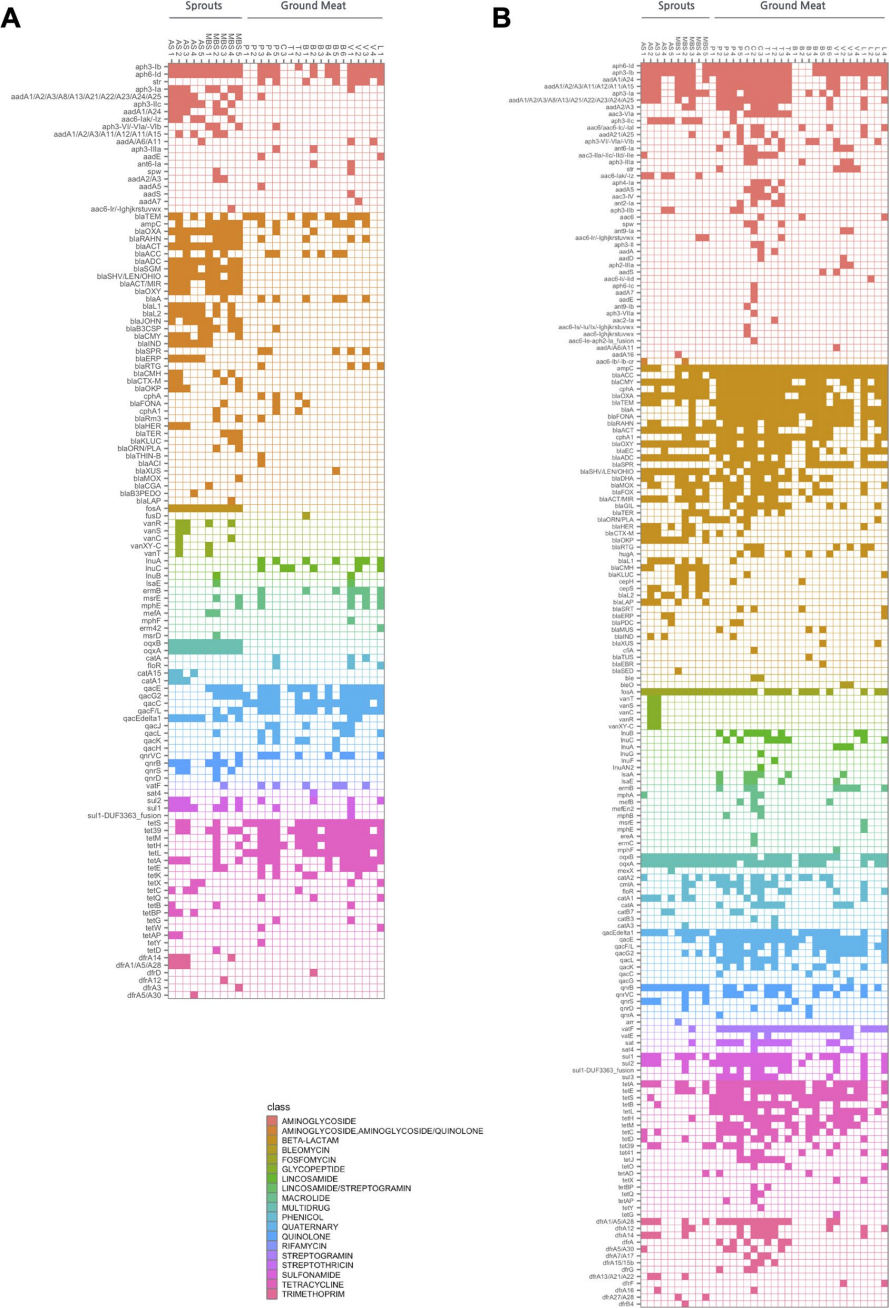
ARG targets detected from retail food commodity samples (**A**) and their corresponding enrichment cultures (**B**) using a targeted metagenomics approach

**Fig. 4:**
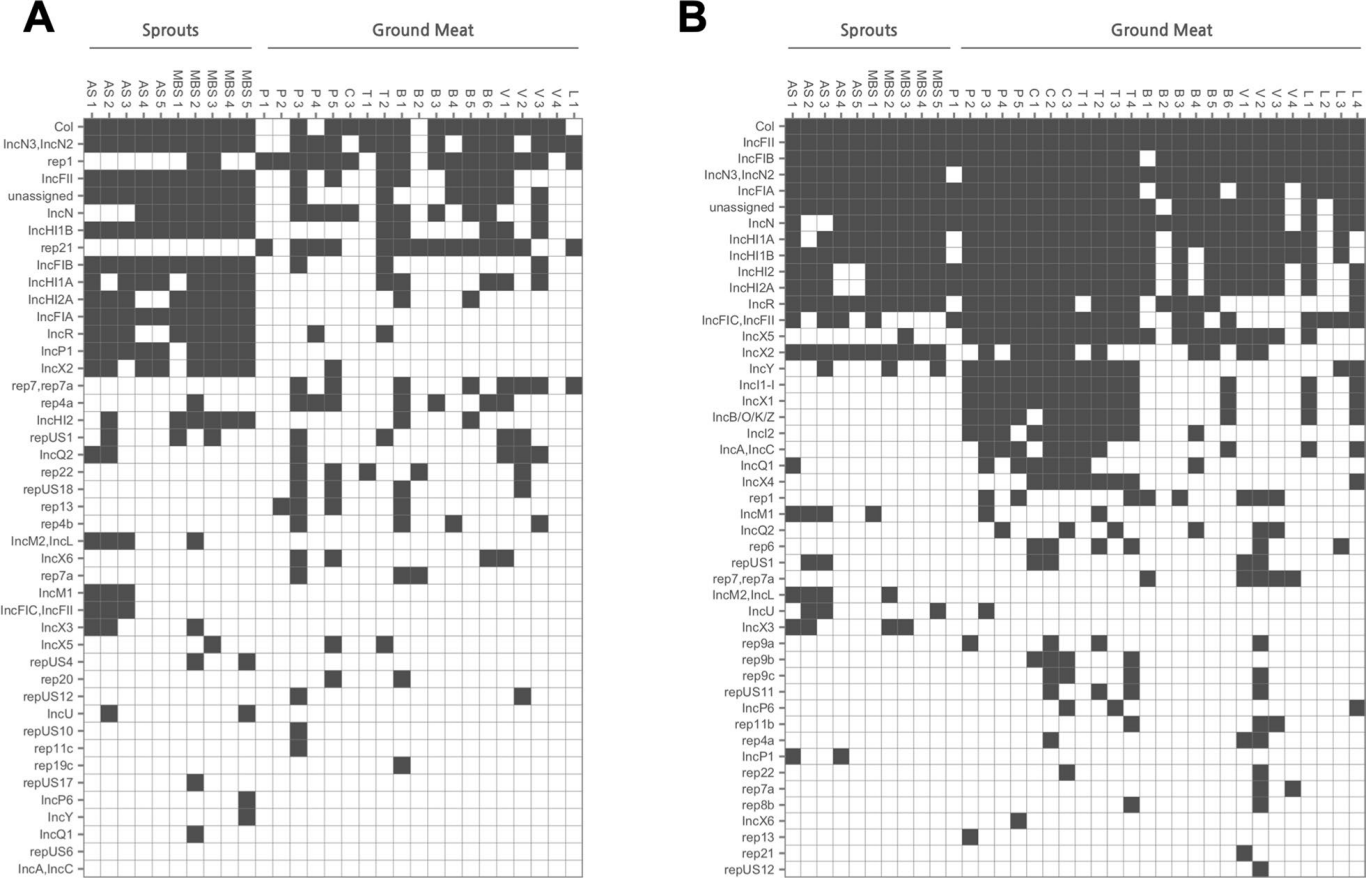
Plasmid replicon targets detected from retail food commodity samples (**A**) and their corresponding enrichment cultures (**B**) using a targeted metagenomics approach

Of the total 72 bait-capture sequencing datasets generated, seven non-enriched food samples were excluded from subsequent analyses due to excessive amount of host nucleic acid found present in their respective metagenomic samples (Additional file 3: Tables S4, S5). For non-enriched food commodity samples, tetracycline resistance genes (including *tetS* and *tetM*) and quaternary ammonium compound resistance genes (e.g. *qacC*) were predominantly detected from the meat samples, whereas beta-lactam resistance genes appeared to be the most prevalent and abundant ARGs found in the sprouts samples (Fig. 3A, Additional file 3: Fig. S4). A noticeably greater variety of ARG targets (especially for those of the aminoglycosides and beta-lactam resistance types) was recovered from the bacterial enrichment samples (Fig. 3B), suggesting the culturally-enriched microbial populations (mostly, bacteria of the *Enterobacteriaceae* family, Additional file 3: Fig. S5) represent significant ARGs reservoirs of the food commodities examined. Similarly, selected replicon targets that were seemingly uncommon among the meat samples but were observed at high frequency with the sprout sample (e.g. replicon types IncFIA, IncHI2A and IncR, Fig. 4A) could be widely detected from the enrichment cultures of both sample categories (Fig. 4B). It is noted that, among the several plasmid clusters detected from the enrichment samples, a small number of replicon types including IncI1-I, IncI2, IncX1, IncX4 and IncB/O/K/Z were exclusively associated with the meat samples, particularly for those belonging to the livestock types of swine (pork) and poultry (chicken, turkey) (Fig. 4B).

**Fig. 5:**
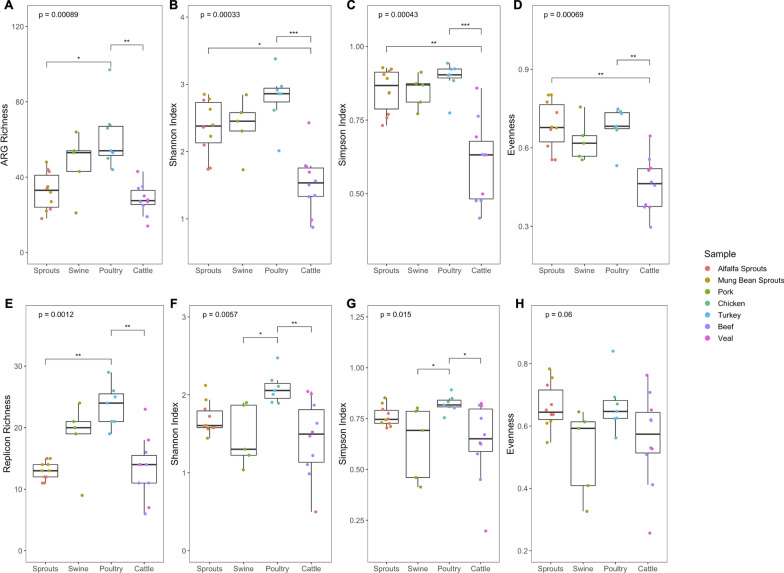
Diversity of ARGs (**A–D**) and replicon (**E–H**) targets detected from food enrichment culture samples. Alpha-diversity indexes were computed based on target detection data derived from host-decontaminated sequencing datasets that were rarefied to 4.2 million read per sample, and were illustrated using box-and-whisker plots with the line inside the box displaying the median value. Samples were colored based on sample types and grouped according to commodity category

Given their ability to survive in the gastrointestinal tract of mammals, and thus become residents therein, *Enterobacteriaceae* bacteria can constitute a particular risk of introducing ARGs into the human gut microbiome. As such, resistome comparisons were conducted using detection data collected from the 36 *Enterobacteriaceae-*enriched food culture samples. Across all 36 food-derived samples, a total of 181 unique AMR genes/gene families of 19 different resistance types, together with 47 unique replicon groups were identified. Of these detected targets, 10 ARGs (namely, *aph6-Id, aph3-Ib, ampC, blaCMY, blaOXA, blaTEM, cphA, oqxB, qacEΔ1,* and, *tetA*) and 10 replicon groups (namely, Col, IncFIA, IncFIB, IncFII, IncHI1A, IncHI1B, IncHI2, IncHI2A, IncN, IncN2/3) were shared by more than half of the samples of each commodity type (Figs. 3B, 4B). Based on the α-diversity indexes computed using both rarefied (Fig. 5) and unrarefied (Additional file 3: Fig. S6) sample datasets, a significant difference was nonetheless observed in the overall resistome diversity between the four commodity types (Kruskal–Wallis test, *p* < 0.001; Fig. 5A–D, Additional file 3: Fig. S6A-D). Richness-wise, poultry samples carried a significantly greater number of unique ARGs on average than samples of cattle and sprouts types (Fig. 5A). Shannon and Simpson diversity indexes, together with Pielou’s evenness, all indicated that the resistome of cattle-type samples possessed significantly lower complexity than those of sprouts and poultry samples (Fig. 5B–D). In regards to replicon diversity, with the exception of evenness, all adopted α-diversity metrics associated with the cattle-type samples differed significantly from those of poultry samples (Fig. 5E–H), reminiscent of the observed difference in their overall resistome diversity (Fig. 5A–D).

**Fig. 6:**
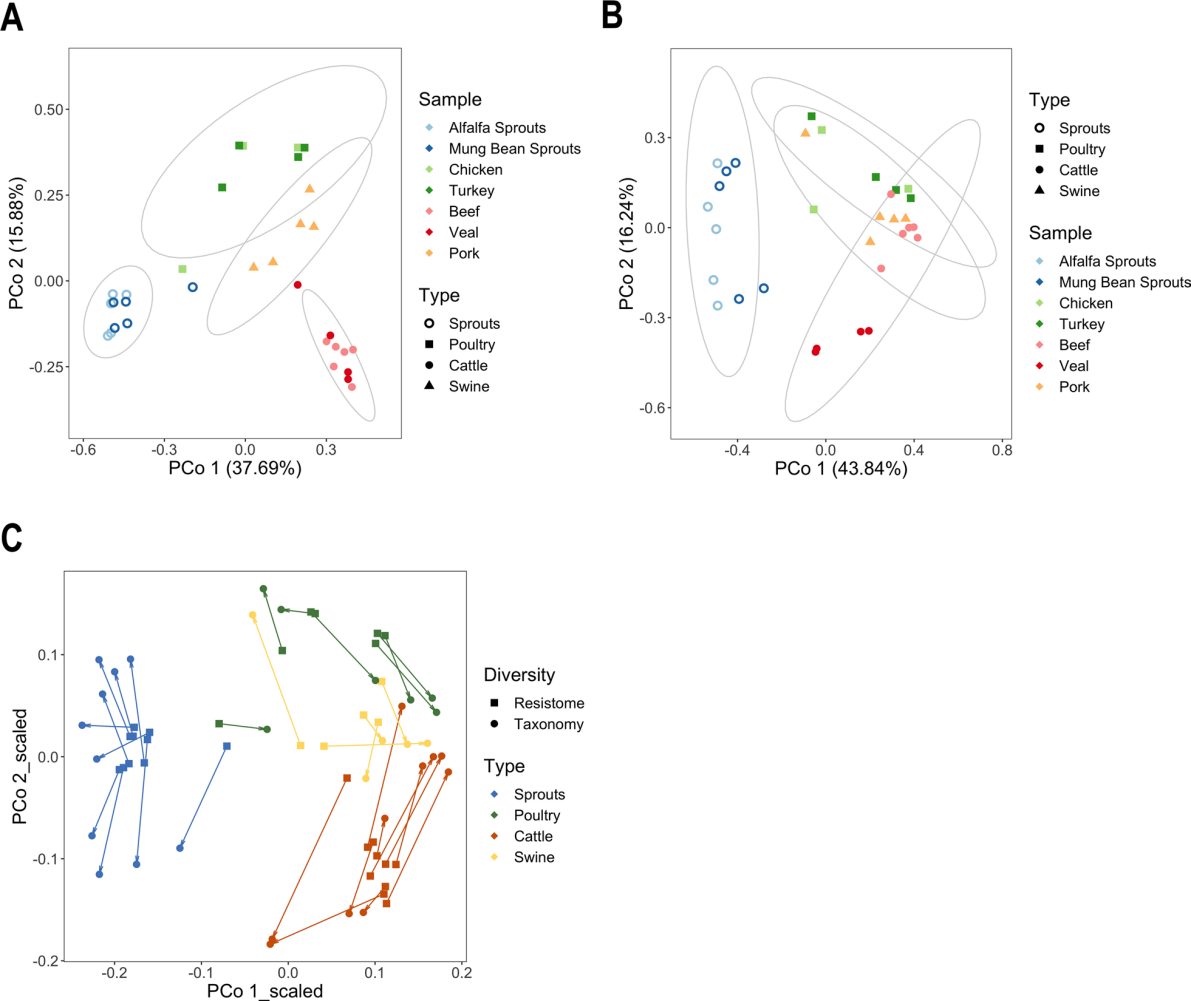
Variations of food-associated resistome profile and microbiota taxonomy. Principle coordinate analysis (PCoA) plots of calculated Bray–Curtis dissimilarities between food enrichment culture samples were constructed based on samples’ ARG profiles (**A**) and genus-level taxonomic composition (**B**). Procrustes analyses (**C**) were conducted to examine the degree to which bacterial and ARG profiles were correlated with each other. Arrows indicate changes in ordination position when resistome (*square*) were compared to the microbiome (*circle*) of individual samples

To further compare the (dis)similarities of resistome profiles and microbiome structures among these samples, principle coordinate analyses (PCoA) based on Bray–Curtis distance were performed. Permutational multivariate analysis of variance (PERMANOVA) detected significant differences in the composition of both resistome (Fig. 6A; *R*^*2*^ = 0.53, *p* = 0.001) and microbiota (Fig. 6B; *R*^*2*^ = 0.48, *p* = 0.001) among the four commodity types, and that more similarities were observed within (than between) commodity types based on analysis of similarities (ANOSIM) statistics (resistome: *R* = 0.85, *p* = 0.001; microbiome: *R* = 0.62, *p* = 0.001). As well, pairwise comparisons revealed that the observed differences between any selected pair of commodity types in terms of their ARG and bacterial compositions were both statistically significant (i.e. adjusted *p* < 0.01), except for the poultry-swine pair (resistome and microbiome) and the cattle-swine pair (microbiome-only). Whereas the ARG profile of sprouts samples displayed less heterogeneity than those of the meat samples, the sprouts-associated resistome was also set apart from the meat-associated resistome, with livestock type explaining most of the ARG variations observed between samples of the poultry, cattle and swine categories (Fig. 6A). Likewise, results obtained from the microbiome PCoA and the corresponding pairwise-comparison statistics also indicated that the meat-derived samples are more similar to each other than to the sprouts’ in terms of their bacterial contents (Fig. 6B). In fact, according to the procrustes analyses performed (Fig. 6C), the observed resistome dissimilarities (but not those of the replicon profile, Additional file 3: Fig. S7) correlated significantly with the taxonomic variation among individual samples (*r* = 0.83, *p* = 0.001). This strongly suggests that the food resistome structure was, to a large extent, dictated by its bacterial composition.

### Taxonomic linkage of detected targets

**Fig. 7:**
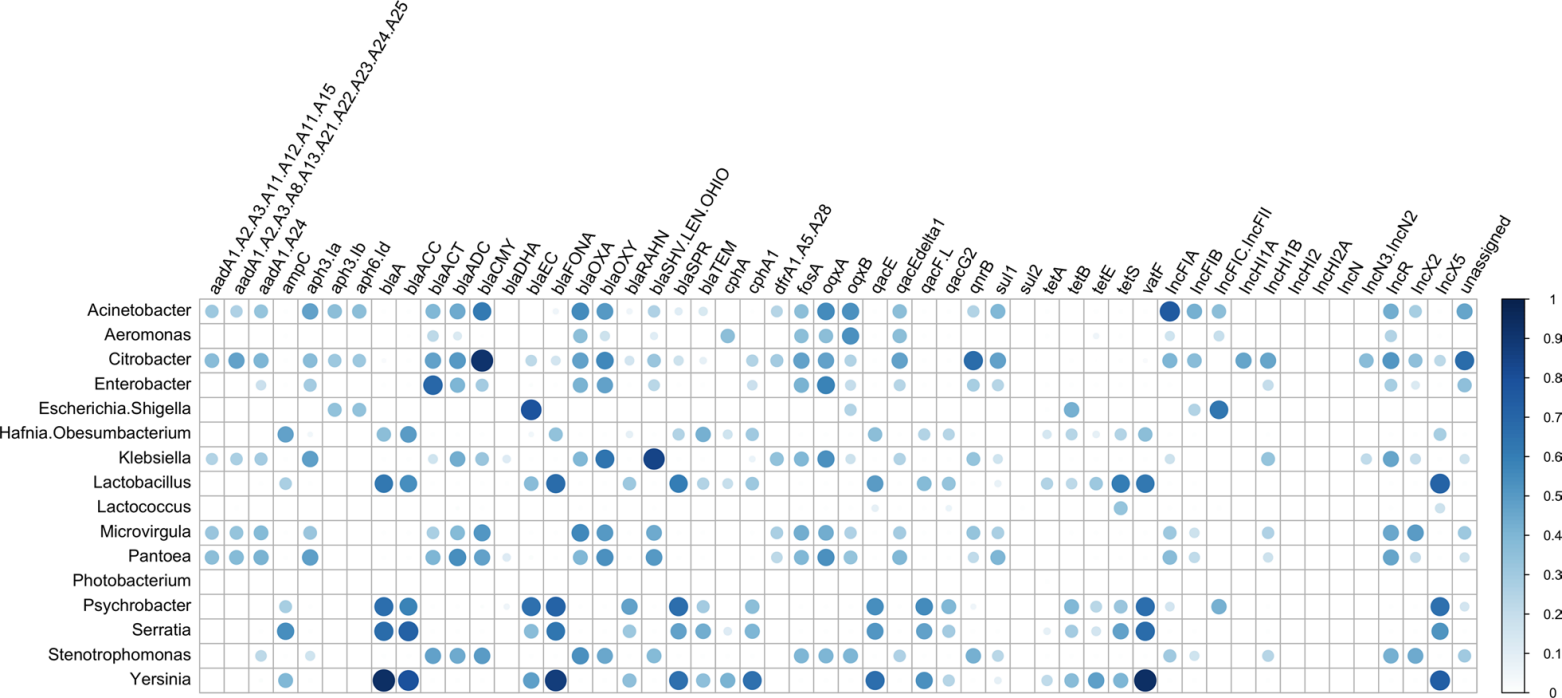
Taxonomic linkage of foodborne ARGs and plasmid replicon types. Correlation matrix of selected bacterial taxa (genus-level) and gene/replicon targets were computed and visualized using R package *corrplot* [47]. Only significant correlations (*p* ≤ 0.01) were shown and depicted by blue circles, sized and shaded based on correlation coefficient values

Co-occurrence of ARG and plasmid replicon targets detected by bait-capture sequencing and representative bacterial taxa revealed by 16S rRNA-based microbial profiling were examined by applying Pearson’s correlation to the binary-transformed subset of data derived from the cultural enrichment samples (Additional file 3: Fig. S8). While there was a general lack of prominent association between the bacterial genera and replicons detected, several strong correlations (*r* ≥ 0.85) were identified among six bacterial genera and seven ARGs (Fig. 7). For instance, beta-lactamase resistance genes *blaA* and *blaFONA* and the streptogramin resistance gene *vatF* were both linked to the genus *Yersinia*; whereas the beta-lactam resistance gene *blaCMY* was frequently detected from samples harbouring *Citrobacter*, implicating these genera as the potential bacterial hosts for the foodborne antibiotic resistance determinants recovered.

## Discussion

Compared to the conventional approach of non-discriminately sequencing the vast metagenome of microbial samples to assess ARG prevalence, this study presents a comprehensive, “resistome-centric” sequencing method that was developed by adapting a sequence-capture/enrichment strategy towards a more sensitive detection of ARG targets. As illustrated using mock bacterial community datasets, our targeted resistome sequencing workflow not only allowed for a near-complete recovery of the known ARG/replicon targets, it did so efficiently with minimal sequencing of extraneous DNA which does not contribute to the resistome profile. The much improved on-target read yield is a favorable outcome of selectively allocating sequencing resources to the pre-captured sequence targets, which also enabled the target detection to be achieved at higher sequence coverage threshold (e.g. 90% cut-off value) without compromising the overall detection sensitivity. This is unlike whole-metagenome shotgun sequencing by which target recovery is only partial and is dependent on both sequencing depth and coverage threshold limit (Additional file 3: Table S3). Hence, within the scope of metagenomic AMR surveillance, it is clear that the herein described target-capture approach offers much better sequencing cost-effectiveness and a greater degree of detection confidence than untargeted shotgun metagenomic profiling method.

Our findings are in agreement with previous studies [21, 22, 48–50] despite using a mock metagenome with different bacterial identity and complexity in the initial validation of our hybridization probe set, which also bears a different blueprint of sequence targets compared to the others’. Once again, it is concluded that the use of a sequence-enrichment strategy in conjunction with massively parallel sequencing can identify more unique ARGs with the use of 5- to tenfold less sequencing resources, and that it is more capable of unveiling the genuine resistome diversity associated with environmental samples. This notion is fully-supported by the comparative retail-food resistome data generated using targeted versus non-targeted sequencing approaches. Specifically, for culturally-enriched and non-enriched samples alike, the bait-capture method consistently revealed a broader spectrum of food resistome elements, of which a substantial portion (up to 97%) was overlooked by the use of whole-metagenome sequencing despite its much greater raw output size (Additional file 3: Fig. S3, Table 2). Although a general improvement in ARG detection from the enrichment samples was observed with the regular shotgun method– a direct result of bacterial enrichment and amplification of their ARG content, the targeted sequencing method provided for further recovery of otherwise-neglected ARGs, and that can be largely attributed to its lower limited of detection (i.e. enhanced sensitivity). A notable observation made from the ground meat samples, especially those belonging to the poultry category, is the overabundance of host DNA sequences found within the metagenomic libraries, both with and without bait-capture (Table 2, Additional file 3: Table S4). Whereas it is evident that our target-capturing system is capable of improving the overall target sequencing efficiency via sequence-enrichment, it is apparently less effective in excluding the non-targeted host nucleic acids when present at high levels in the original metagenomic samples (Table 2). As a limitation of this study, the substantial carry-over of background genetic materials significantly diminished the target sequencing depth of the non-enriched samples, resulting in the under-detection of less-abundant ARG and replicon targets that were otherwise detected from the corresponding enrichment cultures (Figs. 3, 4). Furthermore, we cannot rule out the possibility that the excess amount of eukaryotic DNA contamination had somehow overwhelmed the supposedly specific hybridization process between probes and sequence targets, which could also lead to loss of detection sensitivity. As well, with the use of Illumina’s short-read platform, our targeted-metagenomic approach offers limited sequence coverage of genomic regions beyond the gene targets, resulting in a missed-opportunity for linking ARG to taxonomy through assembly-based methodology. Certainly, while we were able to predict the potential bacterial hosts for some of the detected ARGs via correlation analyses (Fig. 7 and Additional file 3: Fig. S8), cultural-based bacterial isolation and identification of ARG will ultimately be necessary in order to validate the inferred host-ARG relationships. Due to the “targeted” nature of our metagenomic sequencing method, it is to be expected that resistance genes with limited/no sequence homology against the pre-selected gene targets are less likely to be detected. And too, apart from the probe (“bait”) design, the degree of non-/off-target sequences enrichment can also vary depending on the sample matrix and the hybridization reaction conditions. Nonetheless, given the inherent difficulty of eliminating eukaryotic DNA from metagenomic sample preparations, especially for complex sample matrices bearing low bacterial biomass (for example, cow milk [51]), target-capture remains a more pragmatic sequencing approach for evaluating the resistome profile of food samples relative to whole-metagenome sequencing.

Though we are not the first to report on an in-solution hybridization bait-capture system with demonstrated sensitivity and specificity towards diverse ARG targets, it is noteworthy that our bait-set was primarily designed to provide extensive coverage for the reference set of ARGs belonging to NCBI’s National Database of Antibiotic Resistant Organisms database (instead of a more exhaustive ARG database e.g. CARD [27]) with the prime objective of focusing detection on ARGs associated with acquired resistance (i.e. those belonging to a higher risk category). We believe that dedicating almost the entirety of the bait-set to targeting a consortium of well-characterized, pathogen-associated ARGs should ensure a more robust and sensitive detection of AMR genes that are of clinical and evolutionary importance.

Few studies have examined the resistome in various foods in a comprehensive manner. A more complete understanding of the occurrence of ARG-carrying bacteria in foods is needed to assess the impact of this exposure on the spread of AMR. Here, resistome profiles of several high-risk food commodities, including fresh bean sprouts and ground meat, and their respective bacterial enrichment cultures, were successfully obtained using our targeted-metagenomic sequencing workflow. In general, the high occurrence of tetracycline resistance genes observed in retail meat (Fig. 3A, Additional file 3: Fig. S4) is not entirely unexpected, considering that the animal sector represents close to 80% of antimicrobial use in Canada, and that tetracycline is the most distributed antimicrobial agent for use in production animal in both Canada and U.S. [52, 53]. In fact, consistent with our findings, a previous study by Schmdit et al., using a quantitative PCR method, found the *tet(M)* resistance gene in > 93% of 600 samples of U.S. retail ground beef [54]. Another earlier study focused on analyzing AMR in generic *E.coli* recovered from four different types of retail meats acquired in Alberta, Canada, also reported a high prevalence of tetracycline resistance and *tet* genes in the 422 meat isolates examined [55]. On the other hand, it is intriguing, yet somewhat worrisome, that quaternary ammonium compounds (QACs) resistance determinants were readily detected from ground meat samples in the present study (Fig. 3, Additional file 3: Fig. S4). This might be linked to the common practices of using QAC-containing disinfectants in food-processing and food-manufacturing environments for sanitization purposes, which can serve as a strong selection-driver to promote the persistence of QAC resistance-conferring genes among the foodborne microorganisms [56]. Of note, given the small collection of food samples examined in this study, we have yet to observe any detectable level of ARGs that can confer resistance to the last-resort antibiotics colistin/polymyxin (e.g. mcr-1, mcr-2) and carbapenem (e.g. *blaSME*, *blaIMI*, *blaGES*, and *blaKPC*). While the exact bacterial carriers of the beta-lactam resistance genes copiously detected in fresh sprouts microbiota (Fig. 3A, Additional file 3: Fig. S4) remain unknown, they are presumably composed of those selected by the culturing condition used in this study [predominantly, *Gammaproteobacteria* class bacteria (Additional file 3: Fig. S5)] because¸ in most cases, the beta-lactamase-encoding ARGs detected in a sprouts sample were also detected in the corresponding enrichment sample (Fig. 3B and Additional file 3: Fig. S9). Added to this, we have also observed strong correlations between several beta-lactamase genes and bacteria of the *Enterobacteriaceae* and *Yersiniaceae* families detected from these cultured samples (Fig. 7). Collectively, together with the increasingly-common detection of *Enterobacteriaceae* producing various significant beta-lactamases in both environmental- and clinical-settings [57–61], our findings suggest the possibility of exposing the human gut to enteric bacteria harboring significant beta-lactam resistance genes through ingestion of raw sprouts which could have implications on the dissemination of AMR.

Within the context of food safety monitoring, most of the national AMR surveillance activities on retail foods are focused on pathogenic indicator microorganisms and whole genome –based analyses − for instance, the retail meat surveillance components of both the Canadian Integrated Program for Antimicrobial Resistance Surveillance (CIPARS) [62] and the United States’ National Antimicrobial Resistance Monitoring System for Enteric Bacteria (NARMS) [63]. Up until now, the integrative use of metagenomic approaches has not been officially adopted into any of the existing mandated programs, partly attributable to the suboptimal sensitivity of existing methods and the challenges in the harmonization and standardization of metagenomics-based methodology. From a risk assessment point of view, the ability to better report on the presence/absence of high-priority ARGs (and to do so with a greater degree of confidence as we have demonstrated in this study) is a clear merit of the targeted-metagenomic approach. Due to the lack of quantification metrics to evaluate the absolute ARG loads of any selected sample, it is still unclear what to consider as a real risk despite the improved ARG detection sensitivity. As presented in this study, with the often-low bacterial biomass content of food commodity samples and (over-)abundance of host nucleic acid materials present in the food metagenomic DNA samples, a sensible selection of cultural enrichment strategy prior to the application of a sequence-capture approach in conducting targeted resistome sequencing can certainly improve the overall detection of ARGs, especially those coming from priority/significant bacterial members of the food microbiota. However, as a consequence of the use of culture-based procedure during sample preparation, one should be cautious of the potentially-missed components of the total food resistome that could be carried by the unselected and/or non-culturable foodborne bacteria with lower relative abundance.

With the cultured bacteria harboring most of the ARG targets that are present at detectable levels in the non-enriched food samples (Additional file 3: Fig. S9), we speculate that these *Proteobacteria*-dominated populations residing within the food microbiota represent a major reservoir of foodborne ARG. In fact, the significant correlation observed between individual variations of the ARG profile and the bacterial composition among food-derived cultural samples (Fig. 6C) highlights the interrelation between taxonomic diversity and resistome structure, while at the same time, suggesting these culturally-selected foodborne bacteria significantly contribute to the resistome of these high-risk food products. To a certain extent, amongst the three livestock categories examined, we observed a seemingly lower degree of ARG diversity from the cattle-type sample, together with greater resistome similarity among the poultry and swine samples. Although these could be a reflection of the fundamental differences in the ecological development of AMR bacteria within the natural microbiota of food-producing animals, it could also be indicating how the dissemination of ARGs vary under different agricultural settings (e.g. husbandry practices, antibiotics usage, environmental influences). Arguably, under the farm-to-fork concept, the occurrence of ARGs can be attributed to, and impacted by, activities at any stage of the food production continuum—from agriculture and manufacturing to processing and distribution. For example, the detection of certain resistance determinants could be the outcome of meat contamination by AMR bacteria present at the slaughterhouse and/or processing facility that may (or may not) have originated from the meat-producing animal. As such, our resistome profile data of the retail ground meat samples merely captured the gross AMR burden at the consumer level without discerning individual factors that contributed to the overall dynamics of ARGs in the food metagenome.

In conclusion, this study describes the development of a target-capture metagenomic sequencing method and demonstrates its enhanced effectiveness in evaluating the food-associated resistome. Our results not only help fill in the knowledge gap pertaining to human exposure to AMR through retail foods, they also emphasize food as a significant conduit for transmitting AMR genetic determinants of clinical significance.

## Supplementary Information

**Additional file 1.** Sequence targets included in the bait-capture system described in this study.

**Additional file 2.** Raw read filtering metrics for 16s rRNA bacterial profiling sequencing derived from QIIME2.

**Additional file 3.** Supplementary Tables and Figures.
