# Supplementary material for: Exploiting a targeted resistome sequencing approach in assessing antimicrobial resistance in retail foods
Source: Environ Microbiome. 2023 Mar 29;18:25. doi: 10.1186/s40793-023-00482-0 (PMC10052294; doi:10.1186/s40793-023-00482-0)
Supplement: Supplementary file 3 — Additional file 3. Supplementary Tables and Figures. [file 40793_2023_482_MOESM3_ESM.docx]

**SUPPLEMENTAL MATERIALS**

**Table S1. Bacterial composition of a 35-member mock community sample used in this study**

| Bacterial species | | ATCC number |
| --- | --- | --- |
| *Gram positive* | |  |
|  | *Bacillus cereus* | 14579 |
|  | *Listeria innocua* | 33090 |
|  | *Listeria monocytogenes* | 7644 |
|  | *Listeria monocytogenes* | 15313 |
|  | *Staphylococcus warneri* | - |
|  |  |  |
| *Gram negative* | |  |
|  | *Citrobacter freundii* | 8090 |
|  | *Enterobacter cloacae* | 13047 |
|  | *Enterobacter aerogenes* | 13048 |
|  | *Enterobacter aerogenes* | - |
|  | *Escherichia coli* | 35401 |
|  | *Escherichia coli* | 43886 |
|  | *Escherichia coli* | 33849 |
|  | *Escherichia coli* | 31618 |
|  | *Escherichia coli* | 35150 |
|  | *Escherichia coli* | 35218 |
|  | *Escherichia coli (4)* | - |
|  | *Klebsiella pneumoniae* | 13883 |
|  | *Klebsiella oxytoca* | - |
|  | *Proteus mirabilis* | 25933 |
|  | *Pseudomonas aeruginosa* | 10145 |
|  | *Pseudomonas aeruginosa* | 27853 |
|  | *Pseudomonas alcaliphlila (2)* | - |
|  | *Salmonella enterica* | 10723 |
|  | *Salmonella enterica* | 14028 |
|  | *Salmonella enterica* | 8392 |
|  | *Salmonella enterica* | - |
|  | *Serratia marcescens* | 13880 |
|  | *Shigella sonnei* | 29930 |
|  | *Vibrio parahaemolyticus* | 49521 |
|  | *Vibrio parahaemolyticus* | 17802 |
|  | *Yersinia enterocolitica* | 9610 |

**Table S2: Eukaryotic assemblies used for host contamination filtering for different commodities**

| Commodity | Host contaminant genome used (RefSeq) |
| --- | --- |
| Alfalfa sprouts | GCF_000219495.3 [64] |
| Mung bean sprouts | GCF_000741045.1 [65] |
| Ground beef | GCF_002263795.1 |
| Ground veal | GCF_002263795.1 |
| Ground pork | GCF_000003025.6 |
| Ground chicken | GCF_000002315.6 [66] |
| Ground turkey | GCF_000146605.3 [67] |
| Ground lamb | GCF_002742125.1 |

**Table S3. Number of expected target cluster recovered by bait-capture (*Targeted*) and whole-metagenome (*Shotgun*) sequencing from a mock community sample.**

| Subsampling level (million read) | Detection threshold | Shotgun | |  | Targeted^1^ | |
| --- | --- | --- | --- | --- | --- | --- |
|  |  | MC-A | *in silico* |  | MC-A | MC-B |
| 36 | 70% | 100 | 101 |  | - | - |
|  | 80% | 97 | 100 |  | - | - |
|  | 90% | 89 | 93 |  | - | - |
|  |  |  |  |  |  |  |
| 10 | 70% | 83 | 93 |  | - | - |
|  | 80% | 74 | 87 |  | - | - |
|  | 90% | 65 | 70 |  | - | - |
|  |  |  |  |  |  |  |
| 5 | 70% | 66 | 81 |  | 105 | 105 |
|  | 80% | 56 | 64 |  | 104 | 104 |
|  | 90% | 43 | 55 |  | 103 | 103 |
|  |  |  |  |  |  |  |
| 2.5 | 70% | 40 | 53 |  | 105 | 104 |
|  | 80% | 25 | 45 |  | 104 | 104 |
|  | 90% | 18 | 31 |  | 103 | 103 |
|  |  |  |  |  |  |  |
| 1.25 | 70% | 22 | 28 |  | 104 | 104 |
|  | 80% | 12 | 22 |  | 104 | 103 |
|  | 90% | 8 | 14 |  | 103 | 103 |

^1^ Sequencing-depth of the duplicated mock community samples MC-A and MC-B are 8.8 and 5.8 million reads, respectively.

**Table S4. Sequencing metrics of all thirty-six non-enriched food sample data generated using targeted bait-capture sequencing.**

| Sample type | Sample annotation^1^ | Number of raw read | Number of pass quality-filter read | Number of read retained after dehosting | Number of on-target read | Non-host read percentage | On-target rate |
| --- | --- | --- | --- | --- | --- | --- | --- |
| Alfalfa sprouts | AS 1 | 40,624,512 | 37,763,584 | 37,211,044 | 8,108,681 | 99% | 22% |
|  | AS 2 | 21,051,818 | 19,370,424 | 19,316,818 | 3,289,849 | 100% | 17% |
|  | AS 3 | 20,306,092 | 19,767,242 | 19,361,856 | 860,625 | 98% | 4% |
|  | AS 4 | 11,617,334 | 11,281,132 | 10,991,436 | 1,054,279 | 97% | 10% |
|  | AS 5 | 11,964,656 | 11,625,736 | 11,311,324 | 1,411,840 | 97% | 12% |
|  |  |  |  |  |  |  |  |
| Mung bean sprouts | MBS 1 | 11,868,462 | 11,329,122 | 10,872,864 | 659,481 | 96% | 6% |
|  | MBS 2 | 11,149,324 | 10,843,314 | 9,351,052 | 3,112,211 | 86% | 33% |
|  | MBS 3 | 12,648,168 | 12,232,914 | 10,817,564 | 1,561,639 | 88% | 14% |
|  | MBS 4 | 14,091,610 | 13,708,948 | 6,186,158 | 819,934 | 45% | 13% |
|  | MBS 5 | 12,586,252 | 12,260,434 | 8,090,354 | 1,074,279 | 66% | 13% |
|  |  |  |  |  |  |  |  |
| Ground beef | B 1 | 20,299,040 | 19,628,458 | 3,142,362 | 1,983,365 | 16% | 63% |
|  | B 2 | 8,361,590 | 8,110,466 | 2,099,726 | 1,435,389 | 26% | 68% |
|  | B 3 | 16,608,820 | 16,042,812 | 2,790,344 | 1,632,175 | 17% | 58% |
|  | B 4 | 27,689,118 | 25,724,828 | 2,872,674 | 1,750,456 | 11% | 61% |
|  | B 5 | 23,038,874 | 21,741,206 | 2,209,474 | 1,387,074 | 10% | 63% |
|  | B 6 | 7,278,376 | 6,764,134 | 1,814,832 | 711,485 | 27% | 39% |
|  |  |  |  |  |  |  |  |
| Ground veal | V 1 | 8,490,922 | 8,236,600 | 7,673,972 | 3,000,967 | 93% | 39% |
|  | V 2 | 22,919,596 | 22,277,528 | 19,289,230 | 11,658,538 | 87% | 60% |
|  | V 3 | 19,968,500 | 19,446,776 | 16,344,018 | 7,818,450 | 84% | 48% |
|  | V 4 | 10,749,630 | 10,436,562 | 9,875,734 | 1,371,734 | 95% | 14% |
|  |  |  |  |  |  |  |  |
| Ground pork | P 1 | 18,785,910 | 18,042,386 | 209,932 | 121,842 | 1% | 58% |
|  | P 2 | 32,657,936 | 29,975,000 | 1,041,876 | 10,347 | 3% | 1% |
|  | P 3 | 27,982,784 | 25,232,810 | 18,025,568 | 8,585,988 | 71% | 48% |
|  | P 4 | 19,515,606 | 18,683,146 | 1,906,866 | 285,138 | 10% | 15% |
|  | P 5 | 19,244,070 | 18,447,500 | 5,735,896 | 3,254,754 | 31% | 57% |
|  |  |  |  |  |  |  |  |
| Ground chicken | C 1* | 9,776,358 | 9,250,116 | 110,004 | 9,628 | 1% | 9% |
|  | C 2* | 15,118,080 | 14,333,566 | 176,550 | 11,726 | 1% | 7% |
|  | C 3 | 13,357,420 | 12,623,050 | 1,154,064 | 767,215 | 9% | 66% |
|  |  |  |  |  |  |  |  |
| Ground turkey | T 1 | 42,850,468 | 39,869,822 | 1,197,278 | 4,984 | 3% | 0% |
|  | T 2 | 12,868,844 | 12,429,242 | 314,854 | 38,603 | 3% | 12% |
|  | T 3* | 12,118,604 | 11,560,450 | 48,944 | 170 | 0% | 0% |
|  | T 4* | 11,464,990 | 11,037,674 | 67,314 | 418 | 1% | 1% |
|  |  |  |  |  |  |  |  |
| Ground lamb | L 1 | 13,449,338 | 12,836,024 | 975,826 | 193,455 | 8% | 20% |
|  | L 2* | 10,328,254 | 9,916,654 | 17,198 | 211 | 0% | 1% |
|  | L 3* | 10,584,696 | 9,508,940 | 17,082 | 36 | 0% | 0% |
|  | L 4* | 17,781,988 | 17,086,984 | 31,336 | 2,175 | 0% | 7% |

^1^ Asterisks indicate samples excluded from subsequent analyses due to excessive amount of host DNA sequence present.

**Table S5. Sequencing metrics of all thirty six food enrichment culture sample data generated using targeted bait-capture sequencing.**

| Sample type | Sample annotation | Number of raw read | Number of pass quality-filter read | Number of read retained after dehosting | Number of on-target read | Non-host read percentage | On-target rate |
| --- | --- | --- | --- | --- | --- | --- | --- |
| Alfalfa sprouts | AS 1 | 26,466,486 | 24,273,380 | 24,259,486 | 12,042,201 | 100% | 50% |
| (enrichment) | AS 2 | 29,439,020 | 26,885,132 | 26,873,664 | 15,049,566 | 100% | 56% |
|  | AS 3 | 14,963,826 | 14,642,080 | 14,617,954 | 6,805,461 | 100% | 47% |
|  | AS 4 | 11,602,012 | 11,332,460 | 11,192,994 | 4,540,283 | 99% | 41% |
|  | AS 5 | 8,360,430 | 8,170,928 | 8,086,246 | 2,571,700 | 99% | 32% |
|  |  |  |  |  |  |  |  |
| Mung bean sprouts | MBS 1 | 27,706,420 | 25,652,802 | 25,634,690 | 14,296,897 | 100% | 56% |
| (enrichment) | MBS 2 | 13,215,898 | 12,914,110 | 12,812,668 | 7,071,739 | 99% | 55% |
|  | MBS 3 | 32,385,582 | 29,760,326 | 29,742,498 | 16,643,949 | 100% | 56% |
|  | MBS 4 | 15,560,676 | 15,222,888 | 14,824,448 | 8,050,406 | 97% | 54% |
|  | MBS 5 | 15,444,324 | 15,090,562 | 14,939,408 | 6,295,549 | 99% | 42% |
|  |  |  |  |  |  |  |  |
| Ground beef | B 1 | 9,429,730 | 7,768,534 | 7,146,860 | 4,194,769 | 92% | 59% |
| (enrichment) | B 2 | 8,986,674 | 7,451,490 | 7,449,206 | 4,488,268 | 100% | 60% |
|  | B 3 | 17,942,396 | 16,612,778 | 16,559,870 | 8,274,011 | 100% | 50% |
|  | B 4 | 19,673,664 | 17,860,420 | 17,283,810 | 7,899,303 | 97% | 46% |
|  | B 5 | 19,608,260 | 17,778,656 | 17,711,262 | 8,659,495 | 100% | 49% |
|  | B 6 | 8,808,992 | 8,457,692 | 8,068,150 | 4,160,644 | 95% | 52% |
|  |  |  |  |  |  |  |  |
| Ground veal | V 1 | 7,873,228 | 7,663,478 | 7,323,892 | 2,884,494 | 96% | 39% |
| (enrichment) | V 2 | 16,259,880 | 15,776,286 | 15,278,498 | 8,481,576 | 97% | 56% |
|  | V 3 | 18,559,904 | 18,042,998 | 17,301,476 | 6,736,144 | 96% | 39% |
|  | V 4 | 8,579,360 | 8,337,174 | 7,953,762 | 1,350,009 | 95% | 17% |
|  |  |  |  |  |  |  |  |
| Ground pork | P 1 | 4,525,426 | 4,404,560 | 4,233,892 | 1,841,760 | 96% | 44% |
| (enrichment) | P 2 | 29,030,606 | 27,072,554 | 26,274,820 | 14,225,663 | 97% | 54% |
|  | P 3 | 33,343,780 | 29,903,928 | 29,859,086 | 14,236,390 | 100% | 48% |
|  | P 4 | 15,378,942 | 14,864,262 | 14,235,308 | 8,110,779 | 96% | 57% |
|  | P 5 | 8,462,364 | 8,142,016 | 7,793,336 | 3,026,397 | 96% | 39% |
|  |  |  |  |  |  |  |  |
| Ground chicken | C 1 | 25,384,082 | 23,622,722 | 22,476,204 | 13,590,033 | 95% | 60% |
| (enrichment) | C 2 | 13,251,526 | 12,972,768 | 12,614,930 | 8,195,883 | 97% | 65% |
|  | C 3 | 12,692,968 | 12,411,314 | 12,044,198 | 6,320,979 | 97% | 52% |
|  |  |  |  |  |  |  |  |
| Ground turkey | T 1 | 31,401,548 | 29,401,112 | 29,301,744 | 16,849,427 | 100% | 58% |
| (enrichment) | T 2 | 10,614,604 | 10,362,788 | 9,978,492 | 5,401,175 | 96% | 54% |
|  | T 3 | 15,146,362 | 14,819,288 | 14,258,430 | 9,387,108 | 96% | 66% |
|  | T 4 | 17,816,756 | 17,129,930 | 15,633,524 | 9,723,569 | 91% | 62% |
|  |  |  |  |  |  |  |  |
| Ground lamb | L 1 | 13,525,874 | 13,035,790 | 12,298,210 | 5,738,078 | 94% | 47% |
| (enrichment) | L 2 | 11,367,924 | 10,949,106 | 9,151,300 | 4,809,250 | 84% | 53% |
|  | L 3 | 10,796,054 | 10,364,530 | 9,341,806 | 4,659,060 | 90% | 50% |
|  | L 4 | 12,976,316 | 12,476,336 | 12,008,568 | 4,908,345 | 96% | 41% |

**Figure S1. Microbial composition of mock community sample based on metagenomic shotgun sequencing using MetaPhlAn2** [34]**.**

**Figure S2. Food sample types included in this study.** Number in parentheses indicates the number of independent sampling for each food type.

**Figure S3. Comparison of target detection by bait-capture and whole-metagenome sequencings of selected food commodity and cultural enrichment samples**. Numbers in each Venn diagram indicate the number of detected target clusters that is either exclusive to the targeted-sequencing dataset (*purple*) or shared by (*yellow*) both datasets. No target unique to the whole-metagenome sequencing datasets was recovered from any of the fifteen samples.

**Figure S4. Estimated relative abundance of ARGs detected from non-enriched food commodity sample.** Relative abundance was approximated based on proportion of target-mapped reads that was in alignment with ARG target sequences of the specified resistance-type.

**Figure S5. Family-level bacterial taxonomy of cultural enrichment samples (*A*) and their corresponding non-enriched food commodity samples (*B*) included in the targeted-metagenomics analyses.** Each stacked bar represents the average result obtained from up to three independent determination for individual food and enrichment samples using 16S rRNA gene amplicon sequencing.

**Figure S6.** **Diversity of ARGs (*A-D*) and replicon (*E-H*) targets recovered from food enrichment culture samples.** Alpha-diversity indexes were computed based on target detection data generated using non-rarefied sequencing datasets, and were illustrated using box-and-whisker plots with the line inside the box displaying the median value. Samples were colored based on sample types and grouped according to commodity category.

**Figure S7. PCoA of detected replicon profile from food enrichment cultures (*A*) and its relationship with microbiome structure (*B*)**. **A**. Principle coordinate analysis (PCoA) plot of calculated Bray-Curtis dissimilarities between food enrichment culture samples were constructed based on samples’ plasmid replicon profiles (PERMANOVA, *R^2^* = 0.17, *p* = 0.005; ANOSIM, *R* = 0.1464, *p* = 0.014). **B.** Procrustes analysis was conducted to examine the degree to which bacterial and plasmid replicon profiles were correlated with each other. Arrows indicate changes in ordination position when resistome (*square*) were compared to the microbiome (*circle*) of individual samples. (Protest, *r* = 0.30, *p* = 0.11)

**Figure S8.** **Co-occurrence of foodborne bacteria, ARGs, and plasmid replicon type in the culturally-enriched food samples**. Correlation matrix of selected bacterial taxa (genus-level) and gene/replicon targets were computed and visualized using R package *corrplot* [47]. Only significant correlations (*p* ≤ 0.01) were shown and depicted by blue circles that were sized and shaded based on correlation coefficient values.

**Figure S9. ARG targets detected from retail food commodity samples.** A modified illustration based on Figure 3A. Asterisk indicates ARG that was also detected from the corresponding food enrichment culture.


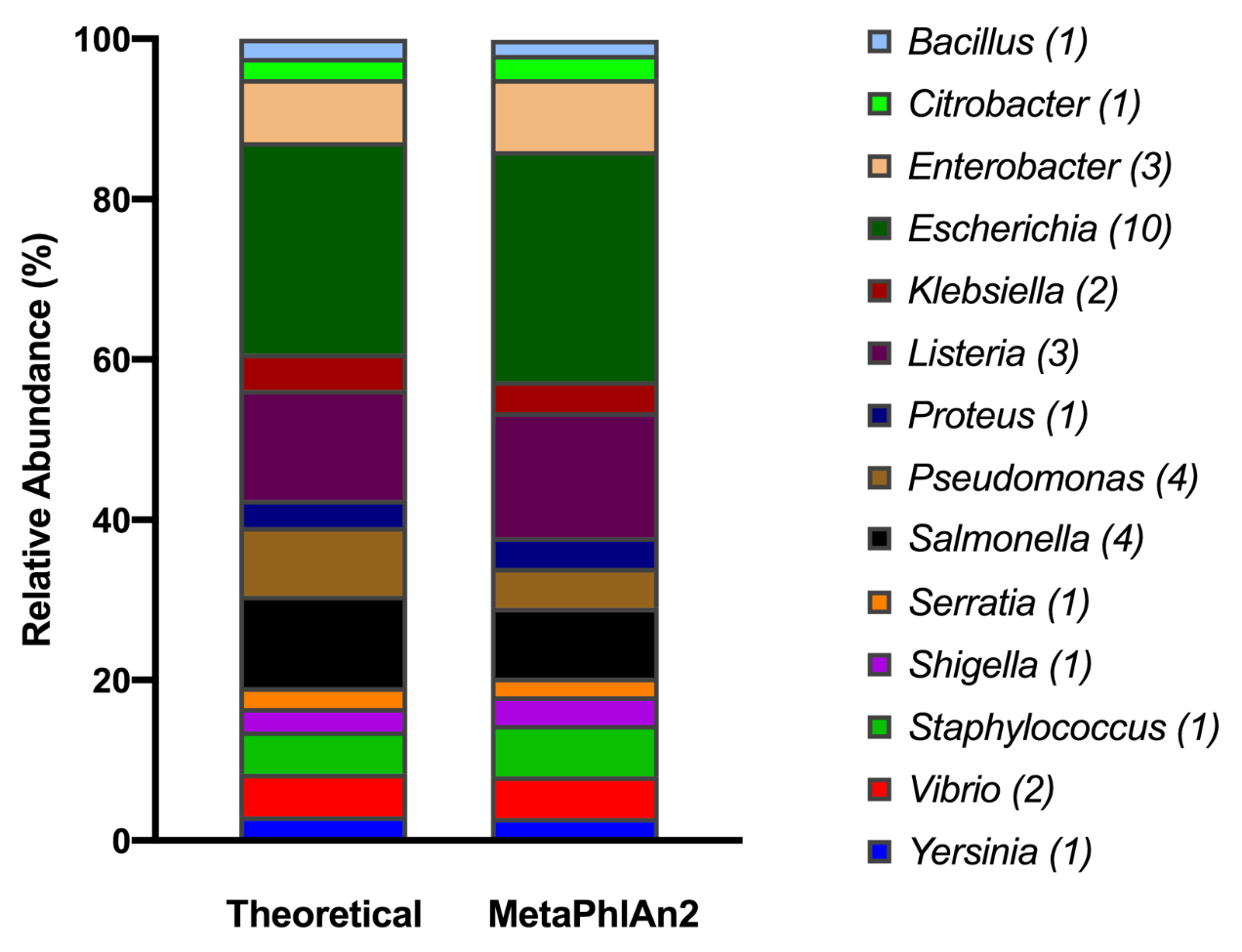


**FIGURE S1**


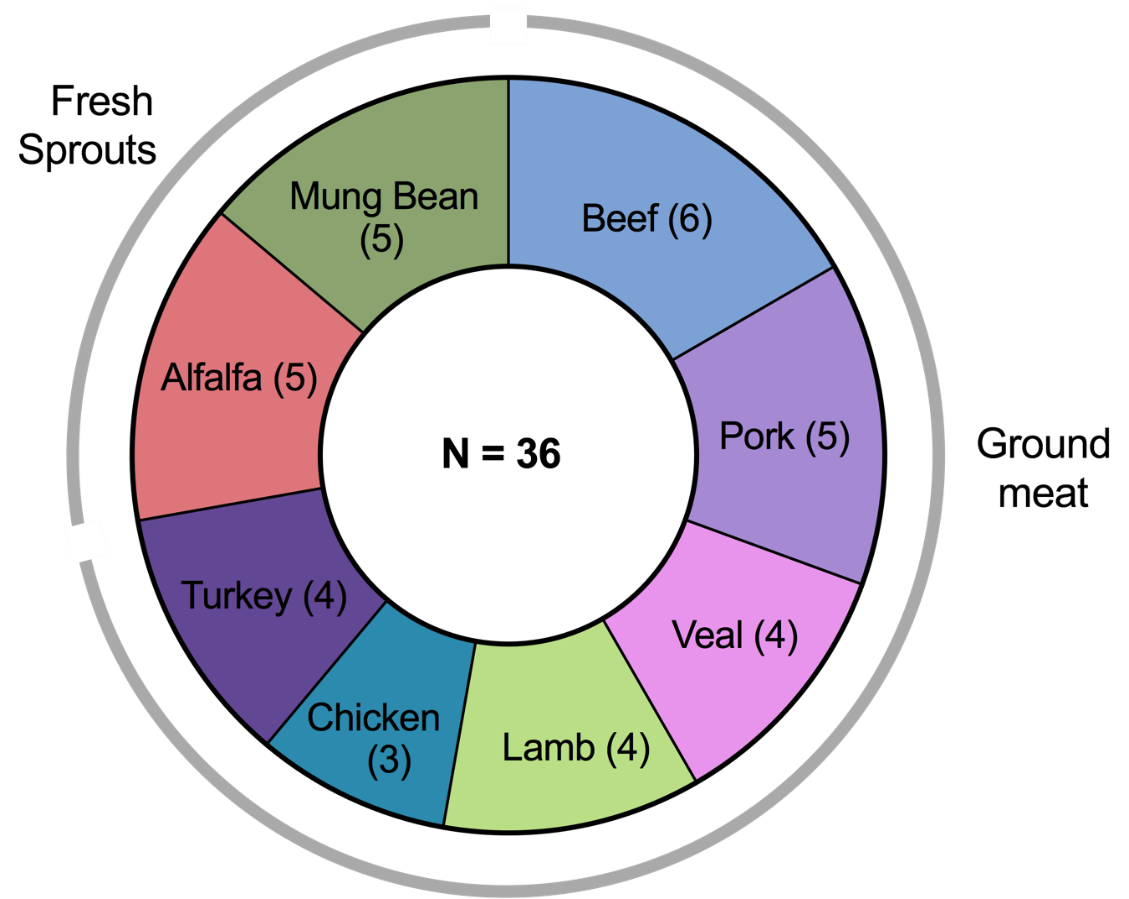


**FIGURE S2**

**
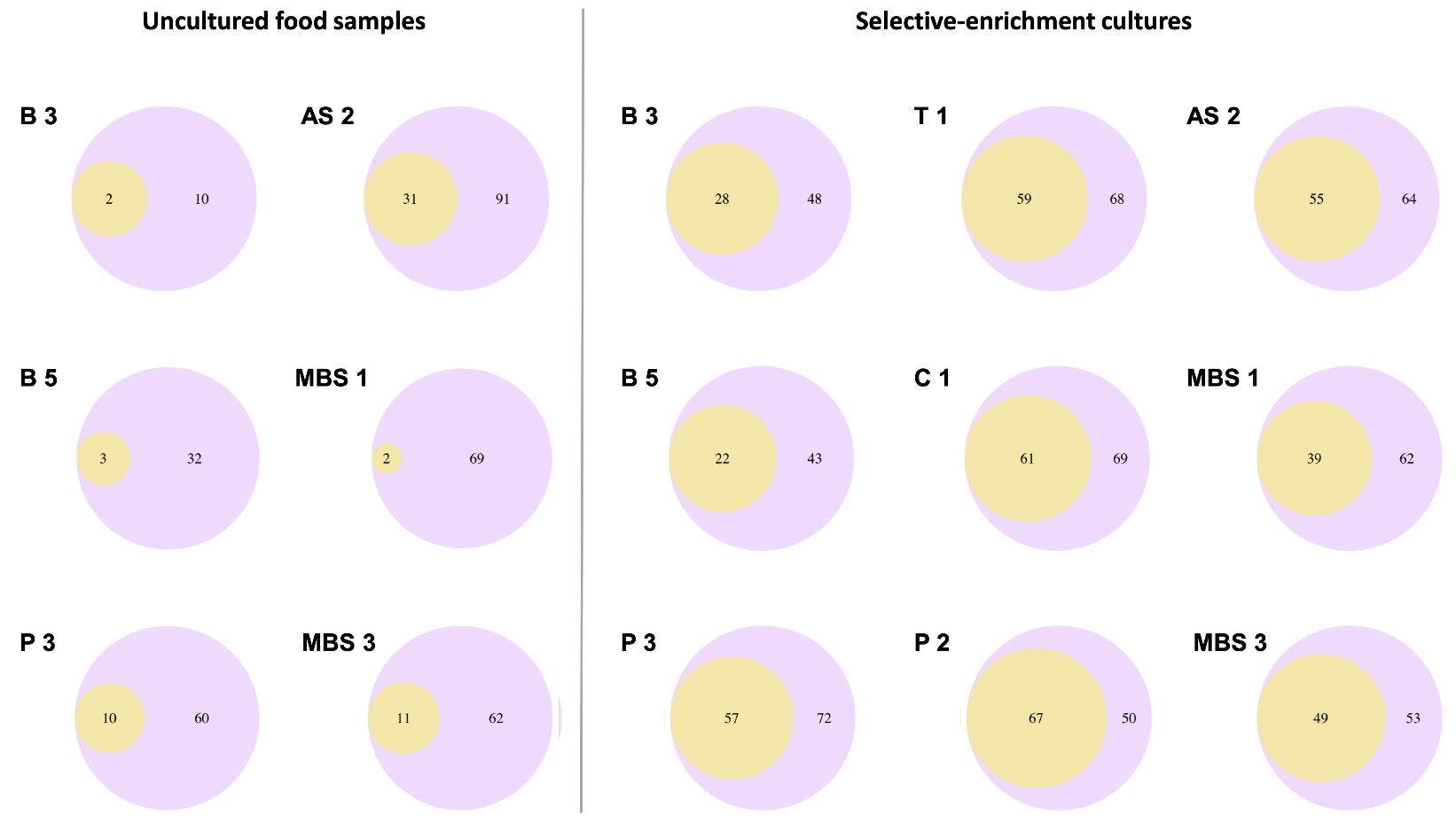
**

**FIGURE S3**

**
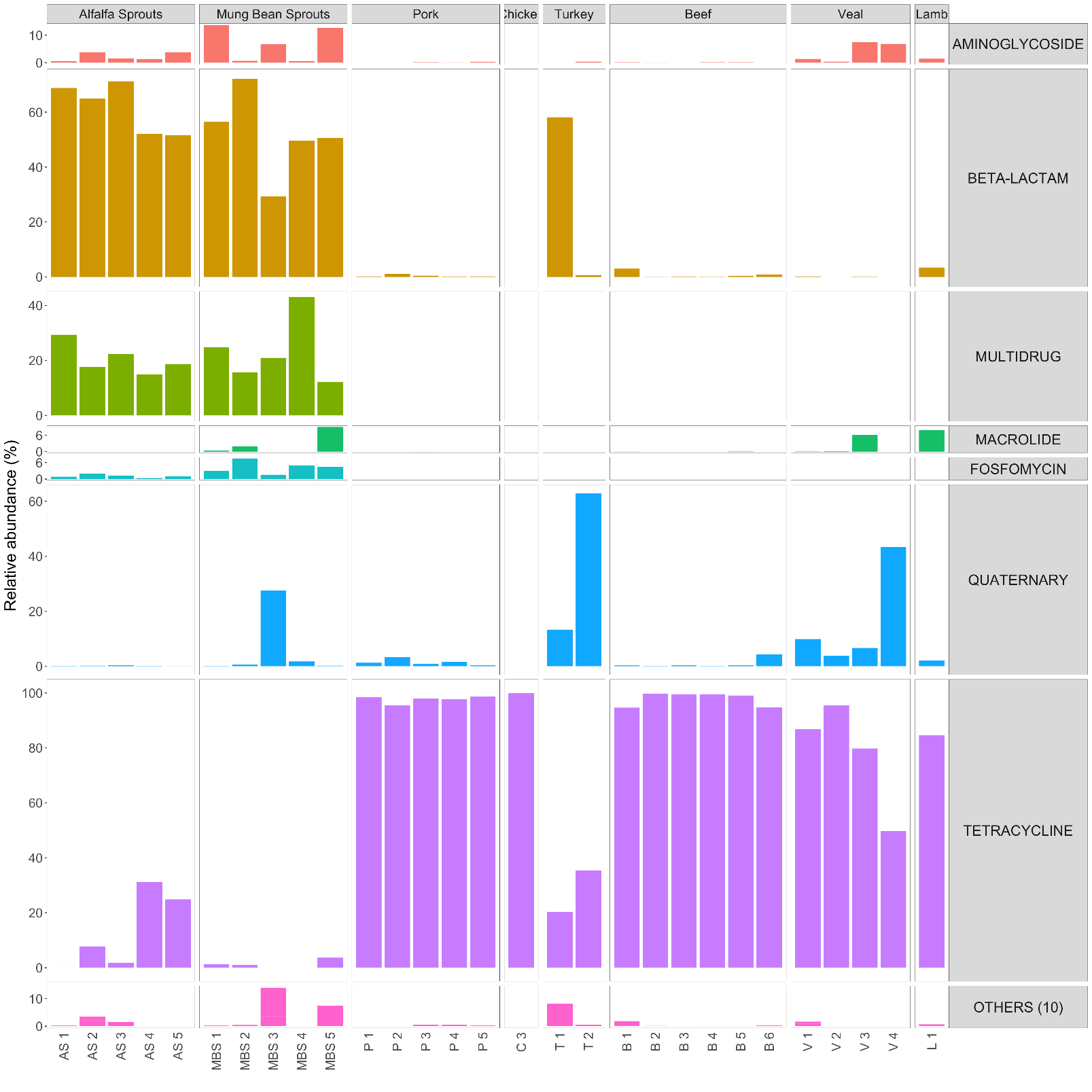
**

**FIGURE S4**

**
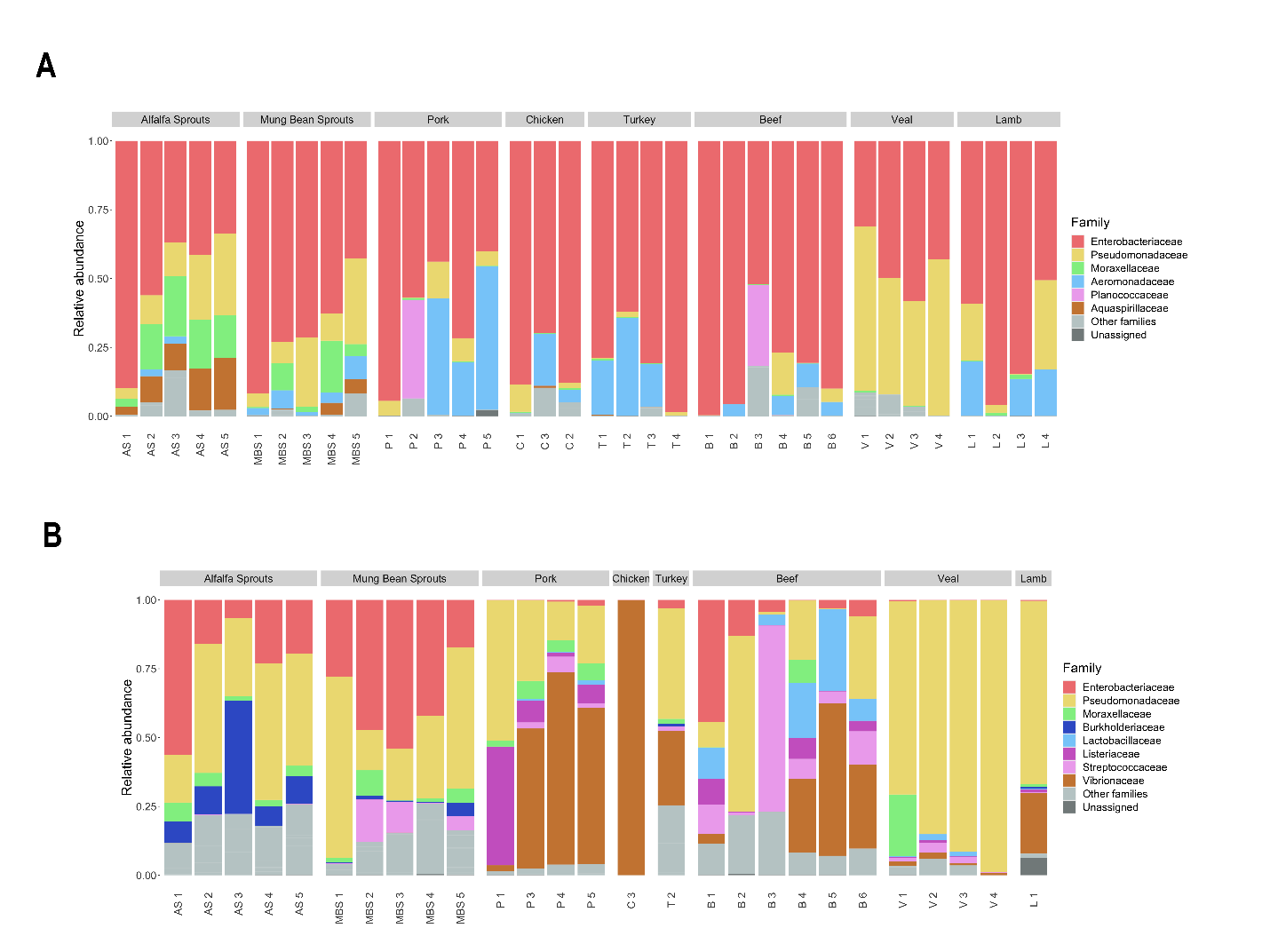
**

**FIGURE S5**

**
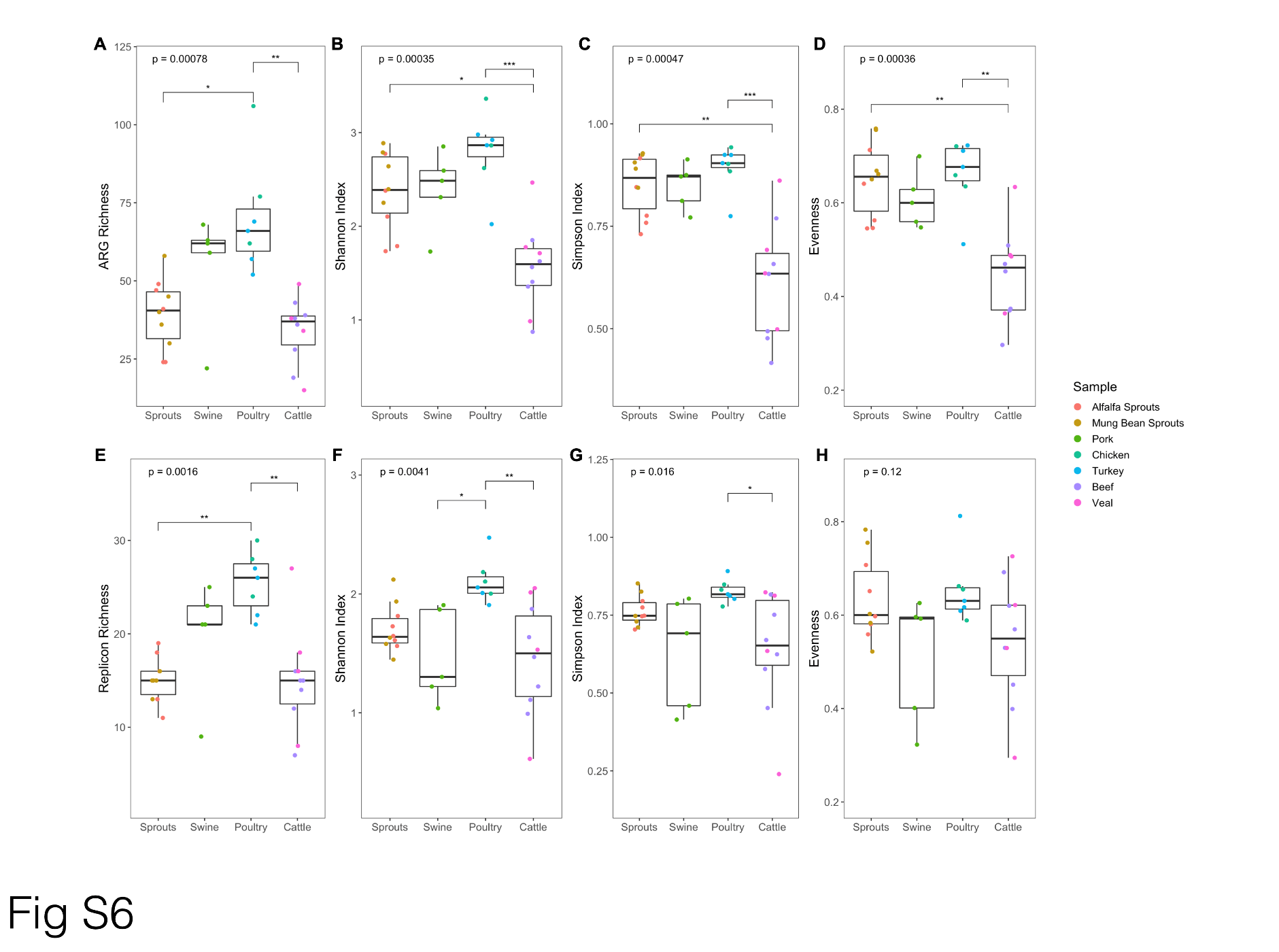
**

**FIGURE S6**

**
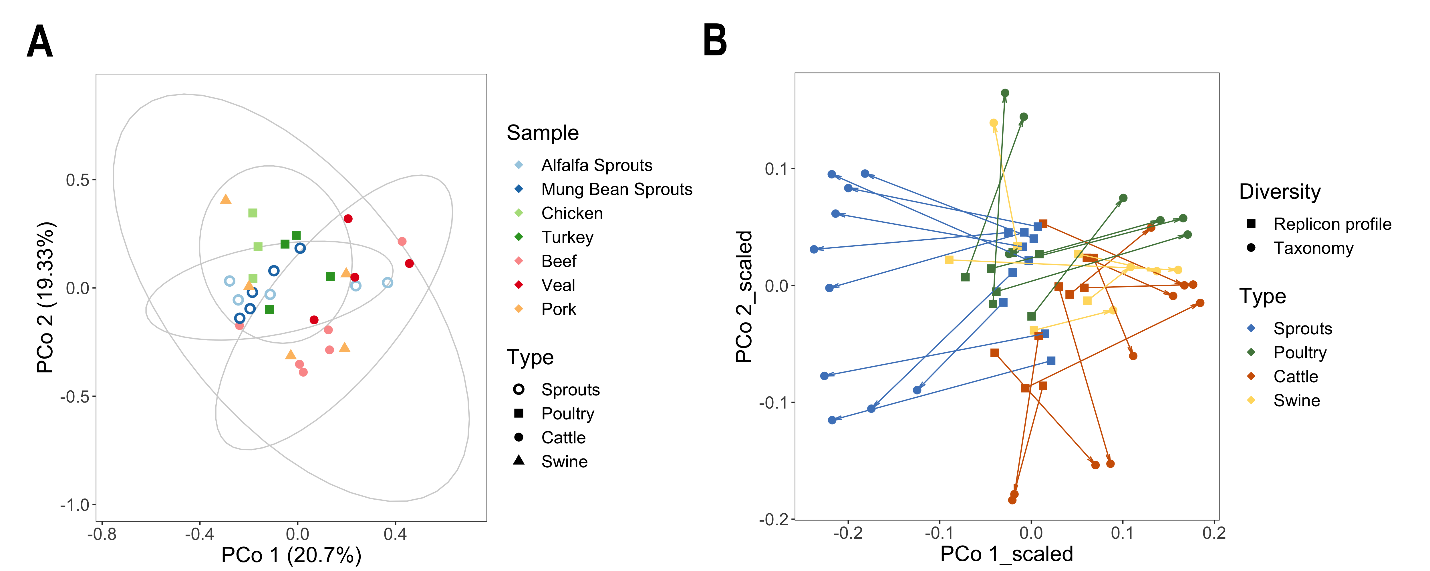
**

**FIGURE S7**

**
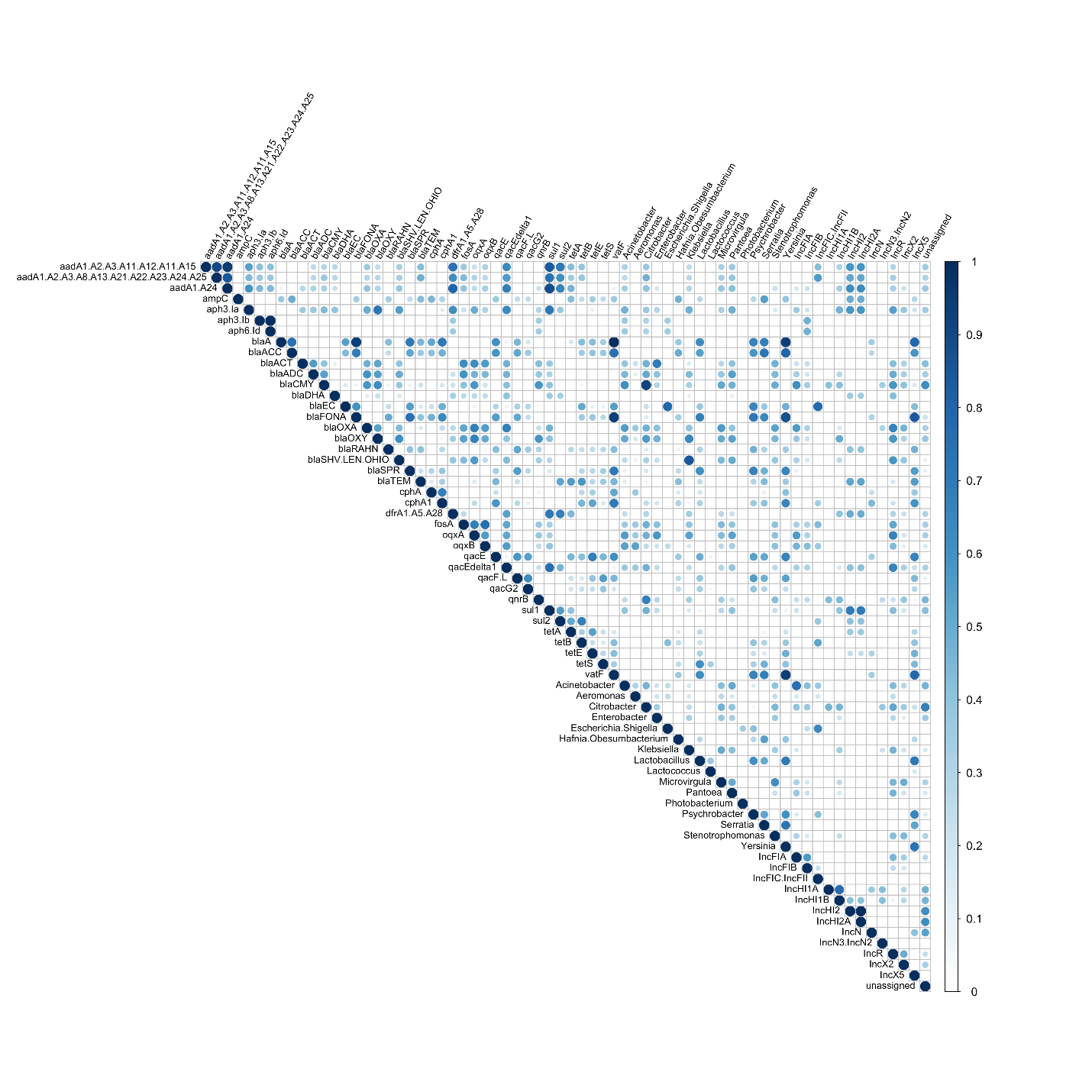
**

**FIGURE S8**

**
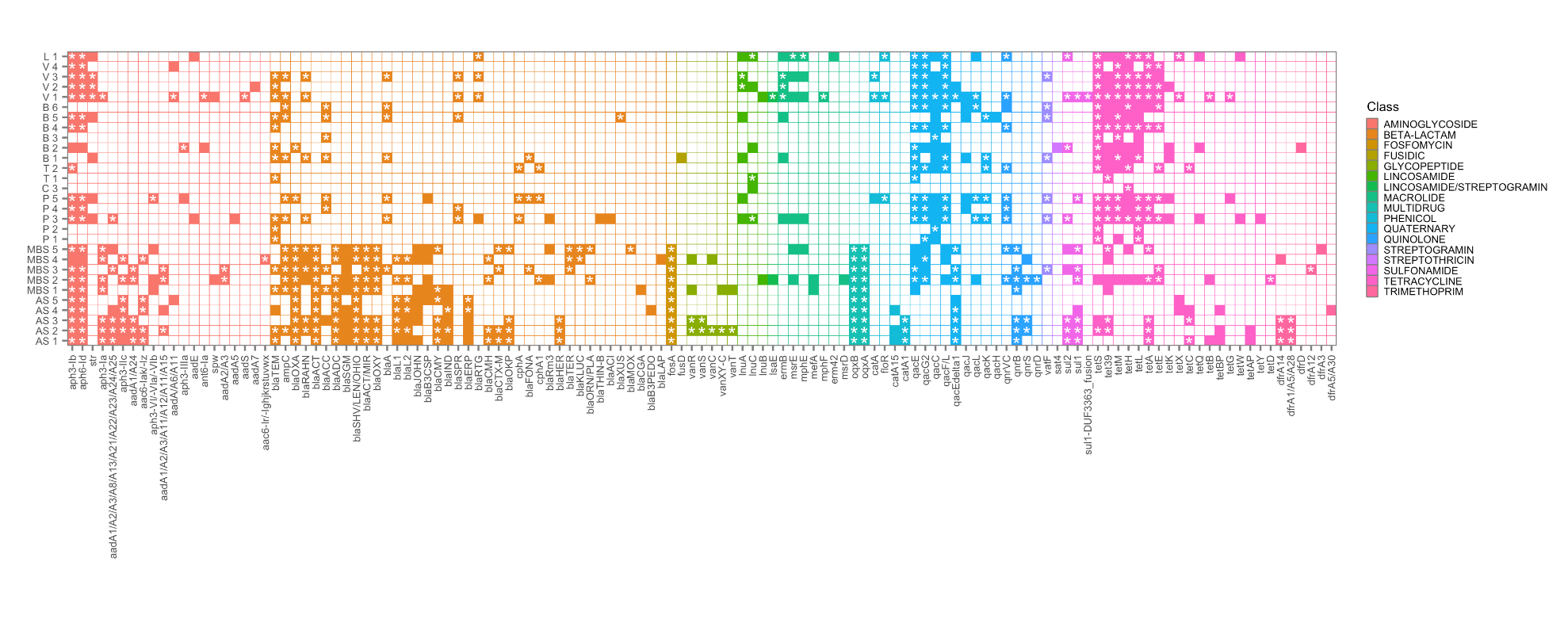
**

**FIGURE S9**
